# Subtype-specific outcome after allogeneic hematopoietic cell transplantation in advanced systemic mastocytosis: a registry study of the European competence network on mastocytosis

**DOI:** 10.1186/s40164-026-00819-8

**Published:** 2026-08-20

**Authors:** Stefan Haug, Wolfgang R. Sperr, Claudia Wehr, Federico Bonofiglio, Saskia K. Klein, Hanneke C. Kluin-Nelemans, André B. Mulder, Olivier Hermine, Julien Rossignol, Felipe Suarez, Ambroise Marcais, William Shomali, Cecelia Perkins, Deborah Christen, Werner Rabitsch, Emir Hadzijusufovic, Jaroslava Voglová, Madlen Jentzsch, Juliana Schwaab, Johannes Lübke, Mattias Mattsson, Hans Hagglund, Ingunn Dybedal, Daniel Baffoe, Patrizia Bonnadonna, Massimiliano Bonifacio, Ilaria Tanasi, Mariarita Sciumè, Marek Niedoszytko, Aleksandar Gorska, Andrzej Mital, Massimo Triggiani, Roberta Parente, Akif Selim Yavuz, Chiara Elena, Luca Malcovati, Jacqueline Ferrari, Friederike Wortmann, Knut Brockow, Tobias Michael Franz, Michael Makris, Sotirios G. Papageorgiou, Christine Breynaert, Toon Ieven, Irena Angelova-Fischer, Alex Stefan, Judit Várkonyi, Vito Sabato, Tanja Daniela Schug, Paul L. A. van Daele, Anna Belloni Fortina, Francesca Caroppo, Theo Gulen, Marc Heizmann, Karin Hartmann, Axel Rüfer, Michael Doubek, Jens Panse, Jason Gotlib, Michel Arock, Hanneke N. G. Oude Elberink, Andreas Reiter, Peter Valent, Khalid Shoumariyeh

**Affiliations:** 1 Institute of Epidemiology and Prevention, Faculty of Medicine, Medical Center-University of Freiburg, Freiburg, Germany; 2 Department of Internal Medicine I, Division of Hematology and Hemostaseology, Medical University of Vienna, Vienna, Austria; 3 Ludwig Boltzmann Institute for Hematology and Oncology, Medical University of Vienna, Vienna, Austria; 4 Department of Medicine I, Medical Center – University of Freiburg, Faculty of Medicine, University of Freiburg, Freiburg, Germany; 5 National Research Council of Italy (CNR), Lerici, Italy; 6 Department of Hematology, University Medical Center Groningen, University of Groningen, Groningen, The Netherlands; 7 Department of Laboratory Medicine, University Medical Center Groningen, University of Groningen, Groningen, The Netherlands; 8 Hôpital Necker-Enfants Malades, APHP, Imagine Institute INSERM U1163, Centre National de Référence des Mastocytoses, Université Paris Cité, Paris, France; 9 Service Hématologie, Hôpitaux Universitaires de Strasbourg, Strasbourg, France; 10 Stanford Cancer Institute/Stanford University School of Medicine, Stanford, CA USA; 11 Department of Oncology, Haematology, Haemostaseology and Stem Cell Transplantation, University Hospital RWTH Aachen, Aachen, Germany; 12 Center for Integrated Oncology, Aachen Bonn Cologne Düsseldorf (CIO ABCD), Aachen, Germany; 13 Department of Internal Medicine I, Bone Marrow Transplantation Unit, Medical University of Vienna, Vienna, Austria; 14 4th Department of Internal Medicine and Hematology, University Hospital Hradec Králové and Charles University, Hradec Králové, Czech Republic; 15 Medizinische Klinik I – Hämatologie, Zelltherapie, Hämostaseologie und Infektiologie, Universitätsklinikum Leipzig AöR, Leipzig, Germany; 16 Department of Hematology and Oncology, University Hospital Mannheim, Heidelberg University, Mannheim, Germany; 17 Department of Medical Sciences, Hematology, Uppsala University, Uppsala, Sweden; 18 Departments of Hematology and Pharmacology, Oslo University Hospital, Rikshospitalet, Oslo Norway; 19 Allergy Unit, Department of Medicine, Azienda Ospedaliera Universitaria Integrata di Verona, Verona, Italy; 20 Department of Engineering for Innovation Medicine, Section of Innovation Biomedicine, Hematology Area, University of Verona, Verona, Italy; 21 S.C. Ematologia, Fondazione IRCCS Ca’ Granda Ospedale Maggiore Policlinico, Milan, Italy; 22 Department of Allergology, Medical University of Gdansk, Gdansk, Poland; 23 Department of Haematology and Transplantology, Medical University of Gdansk, Gdansk, Poland; 24 Division of Allergy and Clinical Immunology, University of Salerno, Salerno, Italy; 25 Division of Hematology, Istanbul Medical School, University of Istanbul, Istanbul, Turkey; 26 Division of Molecular Hematology and Precision Medicine, Fondazione IRCCS Policlinico San Matteo, Pavia, Italy; 27 Department of Hematology and Oncology, University Hospital of Schleswig-Holstein (UKSH) and University Cancer Center of Schleswig Holstein (UCCSH), Lübeck, Germany; 28 Department of Dermatology and Allergy Biederstein, TUM School of Medicine and Health, Technical University of Munich, Munich, Germany; 29 National Center of Expertise for Mastocytosis and Mast Cell Disorders, 2nd Department of Dermatology and Venereology, University General Hospital ‘Attikon’, National and Kapodistrian University of Athens, Athens, Greece; 30 Allergy Unit, 2nd Department of Dermatology and Venereology, University General Hospital ‘Attikon’, National and Kapodistrian University of Athens, Athens, Greece; 31 2nd Department of Internal Medicine, Propaedeutic and Research Institute, Hematology Unit, Attikon University Hospital, University of Athens, Athens, Greece; 32 KU Leuven Department of Microbiology, Immunology and Transplantation, Allergy and Clinical Immunology Research Group and MASTeL, UZ Leuven, Leuven, Belgium; 33 Department of Dermatology, Kepler University Hospital, Johannes Kepler University, Linz, Austria; 34 Department of Internal Medicine, Kepler University Hospital, Johannes Kepler University, Linz, Austria; 35 Department of Hematology, Semmelweis University, Budapest, Hungary; 36 Universiteit Antwerpen, Campus Drie Eiken, Antwerp, Belgium; 37 Univ. Klinik Für Dermatologie, Medical University of Graz, Graz, Austria; 38 Department of Internal Medicine, Erasmus University Rotterdam, Rotterdam, The Netherlands; 39 Department of Immunology, Erasmus University Rotterdam, Rotterdam, The Netherlands; 40 Pediatric Dermatology Unit, Department of Women’s and Children’s Health (SDB), University of Padova, Padova, Italy; 41 Department of Medicine (DIMED), University of Padova, Padova, Italy; 42 Department of Respiratory Medicine and Allergy, Karolinska University Hospital Huddinge, Stockholm, Sweden; 43 Department of Medicine Solna, Karolinska Institutet, Stockholm, Sweden; 44 Division of Oncology, Haematology and Transfusion Medicine, Kantonsspital Aarau, Aarau, Switzerland; 45 Division of Allergy, Department of Dermatology, University Hospital Basel and University of Basel, Basel, Switzerland; 46 Department of Clinical Research, University Hospital Basel and University of Basel, Basel, Switzerland; 47 Department of Biomedicine, University Hospital Basel and University of Basel, Basel, Switzerland; 48 Department of Hematology, Luzerner Kantonsspital, University of Lucerne, Lucerne, Switzerland; 49 University Hospital and Masaryk University, Brno, Czechia; 50 APHP Centre National de Référence Des Mastocytoses, Pitié-Salpêtrière Hospital, University of Sorbonne Paris Cité, Paris, France; 51 Department of Allergology, University Medical Center Groningen, University of Groningen, Groningen, The Netherlands; 52 German Cancer Consortium (DKTK), Partner Site Freiburg and German Cancer Research Center (DKFZ), Heidelberg, Germany

**Keywords:** Allogeneic stem cell transplantation, Systemic mastocytosis, Advanced systemic mastocytosis, Tyrosine kinase inhibitors, *KIT* D816V, IPSM, MARS

## Abstract

Advanced systemic mastocytosis (AdvSM), comprising aggressive systemic mastocytosis, mast cell leukemia, and systemic mastocytosis with an associated hematological neoplasm, is a heterogeneous myeloid neoplasm with poor prognosis. Allogeneic hematopoietic cell transplantation (allo-HCT) remains the only potentially curative treatment, yet its benefit across AdvSM subtypes in the era of KIT-targeted tyrosine kinase inhibitors (TKIs) such as midostaurin and avapritinib remains unclear. To identify patient subgroups benefiting from allo-HCT and to define prognostic factors for post-transplant survival, we analyzed 631 AdvSM patients from the European Competence Network on Mastocytosis registry, including 69 who underwent allo-HCT. Treatment effects were assessed using time-dependent Cox regression in a transplant-eligible complete-case cohort (*n* = 419). In this cohort, allo-HCT showed no overall survival (OS) benefit (hazard ratio [HR] 1.21, 95% CI 0.80–1.84; *p* = 0.37), whereas TKI response emerged as a strong independent predictor of survival (HR 0.42; *p*<0.001). Subtype-stratified analysis revealed an allo-HCT benefit exclusively in patients with systemic mastocytosis associated with acute myeloid leukemia (*n *= 30; HR 0.20; *p* = 0.020). Among the 69 transplanted patients, median OS was 49.6 months with 2-year and 5-year survival of 63% and 46%, respectively. The International Prognostic Scoring System for Mastocytosis (IPSM) at transplantation independently predicted both OS (HR per category 1.68; *p* = 0.026) and progression-free survival (HR 1.99; *p* = 0.003), whereas the Mutation-Adjusted Risk Score and diagnostic subtype did not reach statistical significance. Competing risk analysis demonstrated that higher IPSM captured both relapse-related and transplant-related mortality. These findings suggest that the survival benefit of allo-HCT in AdvSM is driven primarily by control of the associated myeloid neoplasm. Accordingly, allo-HCT should be prioritized in patients with SM-AML, whereas TKI-directed strategies may be preferred in other subtypes, with IPSM at transplantation potentially guiding transplant selection and timing.

**Supplementary Information:**

The online version contains supplementary material available at 10.1186/s40164-026-00819-8.

To the editor,

Advanced systemic mastocytosis (AdvSM), comprising aggressive SM (ASM), SM with an associated hematological neoplasm (SM-AHN), and mast cell leukemia (MCL), is a rare *KIT*-driven myeloid neoplasm with poor prognosis [1, 2]. Allogeneic hematopoietic cell transplantation (allo-HCT) remains the only potentially curative treatment, yet its survival benefit has never been compared against modern non-transplant strategies in the era of KIT-targeting tyrosine kinase inhibitors (TKIs) such as midostaurin and avapritinib [3, 4]. Prior allo-HCT analyses were limited by pre-TKI data, lack of prognostic stratification, or absence of non-transplanted comparators [5–7]. Whether allo-HCT improves survival across AdvSM subtypes, and which patients benefit most, remains unresolved.

We analyzed the European Competence Network on Mastocytosis (ECNM) registry, including 631 AdvSM patients diagnosed between 1988 and 2022 from 32 centers across 16 countries, of whom 69 (11%) underwent allo-HCT [8]. To assess treatment effects while accounting for confounders, we restricted comparative analyses to a transplant-eligible complete-case cohort (*n* = 419; age 18–75 years, WHO performance status ≤3, complete prognostic data), of whom 227 (54%) received TKI treatment, and 63 (15%) were transplanted, modeling allo-HCT and TKI response as time-dependent covariates. Sensitivity analyses confirmed negligible immortal-time bias. The International Prognostic Scoring System for Mastocytosis (IPSM) and Mutation-Adjusted Risk Score (MARS) were evaluated for post-transplant risk stratification [9, 10]. Detailed methods, baseline characteristics, and additional analyses are provided in the supplementary appendix.

Our study yields three principal findings with potential clinical implications. First, allo-HCT did not confer a uniform survival benefit across AdvSM subtypes in a multivariate analysis (HR 1.21, 95% CI 0.80–1.84; *p* = 0.369). A benefit was observed only in systemic mastocytosis with associated acute myeloid leukemia (SM-AML; HR 0.20, 95% CI 0.05–0.78; *p* = 0.020), with no benefit in ASM±AHN (HR 1.04; 95% CI 0.50–2.17; *p* = 0.911), MCL±AHN (HR 1.20; 95% CI 0.48–2.99; *p* = 0.70), or other SM-AHN (HR 2.02; 95% CI 0.71–5.81; *p* = 0.19) (Fig. 1, supplementary appendix). Given the limited size of the SM-AML subgroup, this estimate must be regarded as exploratory. These findings nonetheless support subtype-specific patient selection, and align with the concept that the survival benefit of allo-HCT in AdvSM is driven primarily by control of the associated myeloid neoplasm rather than eradication of clonal mast cell disease alone [11].

**Fig. 1:**
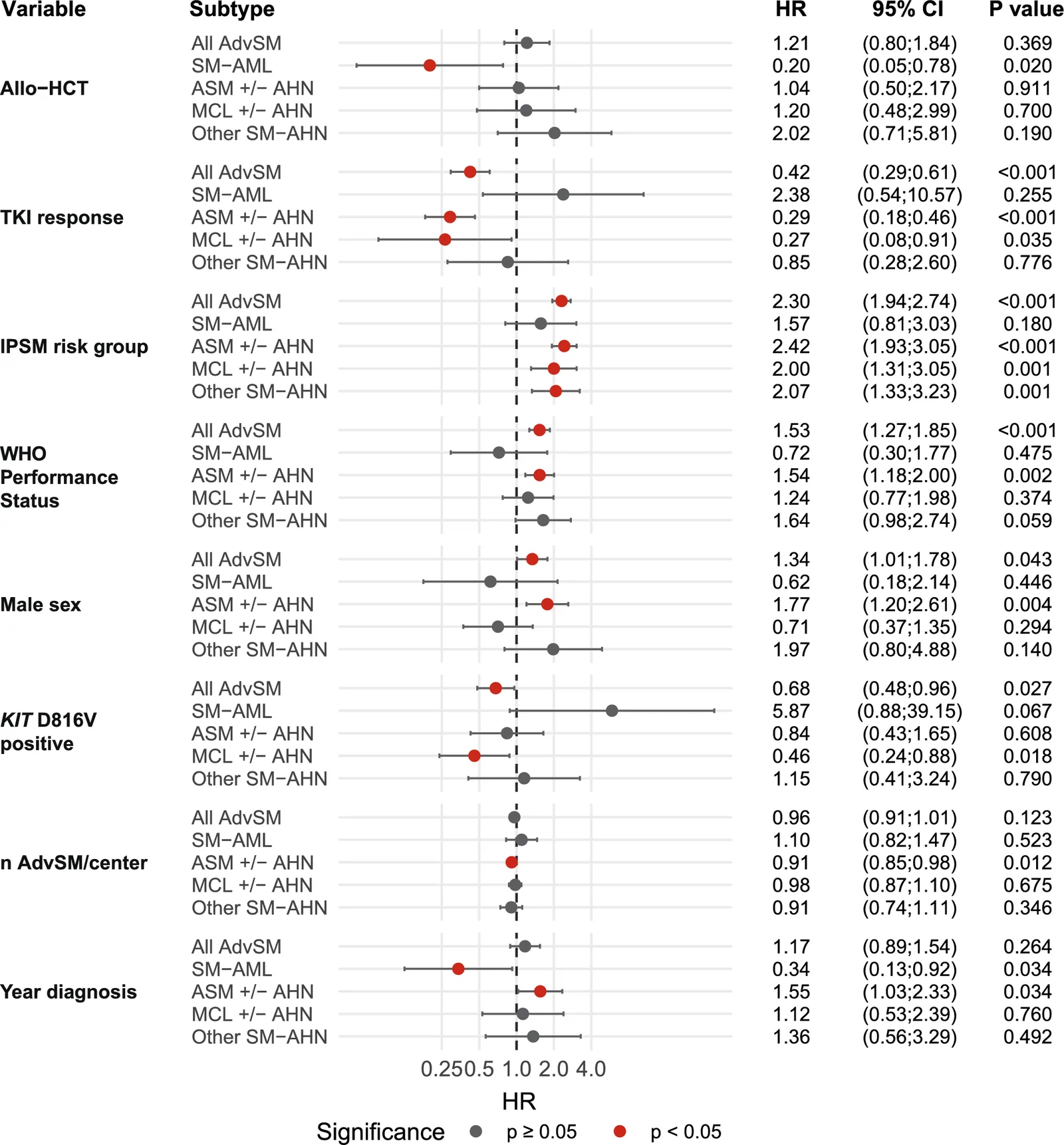
Subtype-stratified multivariate analysis of prognostic factors for overall survival in AdvSM. Forest plot from the multivariate Cox regression stratified by AdvSM subtype (complete-case cohort, *n = 419*). "All AdvSM" shows the analysis for the unstratified cohort. Hazard ratios (HRs) > 1 indicate increased mortality risk. Allo-HCT and TKI response were modeled as time-dependent covariates; other covariates include IPSM risk group (per category), WHO performance status (per point), sex, *KIT* D816V status, center experience (per 10 patients), and year of diagnosis (per decade). Within the complete-case cohort, 227 patients (54%) received TKI treatment and 63 (15%) received allo-HCT. Abbreviations: AdvSM, advanced systemic mastocytosis; AHN, associated hematologic neoplasm; allo-HCT, allogeneic hematopoietic cell transplantation; ASM, aggressive systemic mastocytosis; CI, confidence interval; HR, hazard ratio; IPSM, International Prognostic Scoring System for Mastocytosis; MCL, mast cell leukemia; n, number; SM-AHN, systemic mastocytosis with an associated hematological neoplasm; SM-AML, systemic mastocytosis with an associated acute myeloid leukemia; TKI, tyrosine kinase inhibitor; WHO, World Health Organization

Second, TKI response emerged as a strong independent predictor of overall survival (OS, HR 0.42, 95% CI 0.29–0.61; *p* < 0.001), with significant benefit in ASM±AHN (HR 0.29; 95% CI 0.18–0.46; *p* < 0.001) and MCL±AHN (HR 0.27; 95% CI 0.08–0.91; *p* = 0.035) (Fig. 1). This supports a central role for TKI-directed therapy in patients without associated AML.

Third, we provide the first validation of IPSM at transplantation as an effective tool for post-transplant risk stratification. Among 69 transplanted patients (median post-transplant follow-up 26.2 months; median OS 49.6 months; 2-year OS 63%, 5-year OS 46%), higher IPSM at transplantation independently predicted both worse OS (HR per category 1.68, 95% CI 1.07–2.65; *p *= 0.026) and worse progression-free survival (PFS) (HR per category 1.99, 95% CI 1.27–3.11; *p *= 0.003), outperforming both MARS (*n* = 43, non-significant trends only) and diagnostic subtype classification. When stratified into favorable (IPSM 1–2) versus unfavorable (IPSM 3–4) categories, 2-year OS was 72% versus 48% and 2-year PFS 60% versus 32% (log-rank *p *< 0.01 for both) (Fig. 2). All 7 patients in IPSM group 4 relapsed within 12 months and died within 36 months. Competing risk analysis demonstrated that IPSM captures both relapse-related (5-year: 33% for groups 3–4 vs 22% for groups 1–2) and transplant-related mortality (26% vs 14%), providing risk stratification at transplant planning.

**Fig. 2:**
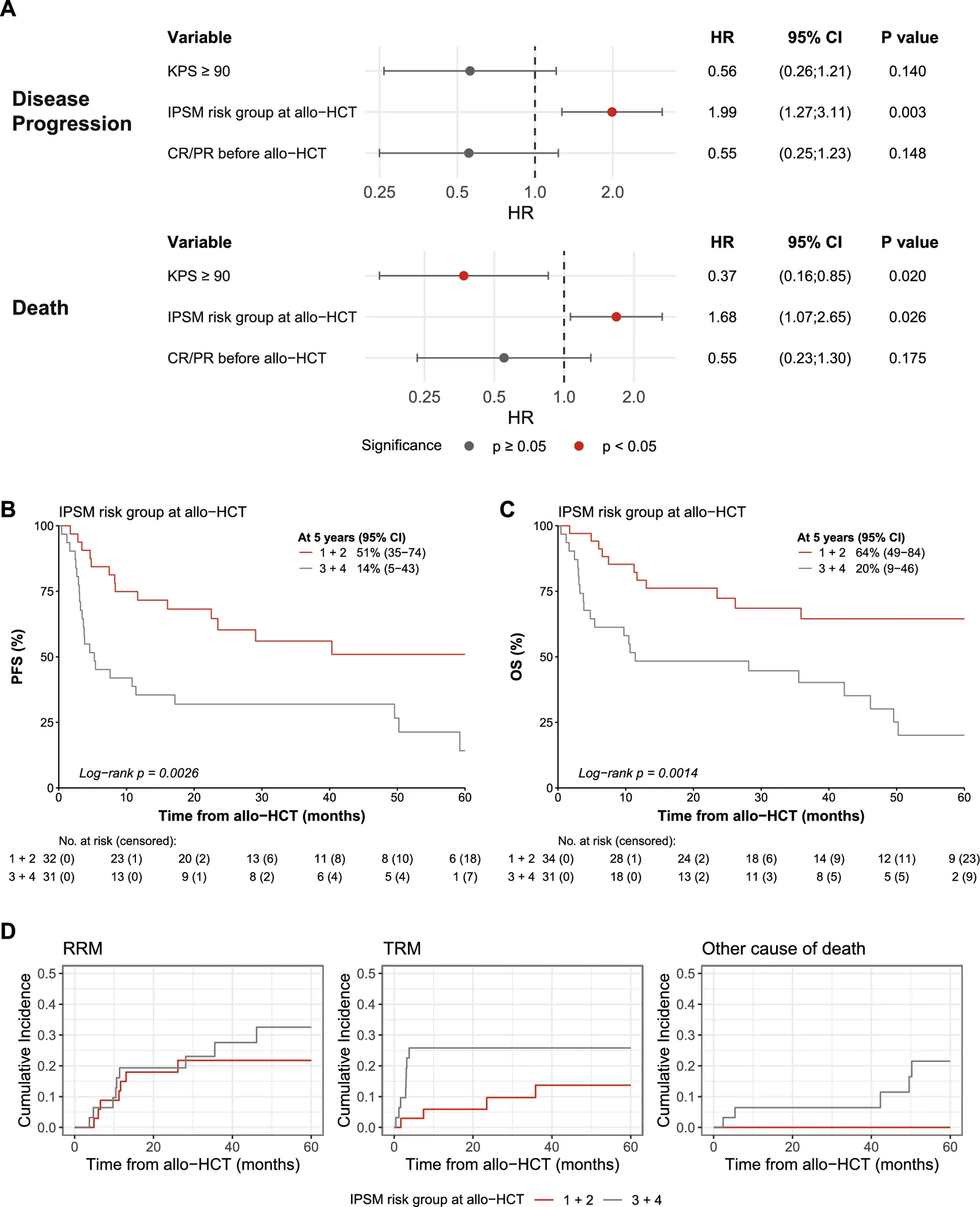
Prognostic factors for overall and progression-free survival after allo-HCT in AdvSM. **A** Forest plot from multivariate analysis of AdvSM patients treated with allo-HCT, showing hazard ratios (HRs) for disease progression (top panel) and death (bottom panel) after allo-HCT. HR > 1 indicates increased risk for progression or death. Only patients with complete covariate data were included (*n* = 50 for disease progression; *n* = *52* for survival). IPSM risk group after conditioning was modeled per 1-category increase. **B** Kaplan–Meier estimates of progression-free survival (PFS) after allo-HCT stratified by combined IPSM risk groups after conditioning (IPSM 1 + 2 vs. IPSM 3 + 4). **C** Kaplan–Meier estimates of overall survival (OS) after allo-HCT stratified by combined IPSM risk groups after conditioning (IPSM 1 + 2 vs. IPSM 3 + 4). **D** Cumulative incidence of relapse-related mortality (RRM), transplant-related mortality (TRM), and other causes of death stratified by combined IPSM risk groups after conditioning. Abbreviations: AdvSM, advanced systemic mastocytosis; allo-HCT, allogeneic hematopoietic cell transplantation; CI, confidence interval; CR, complete remission; HR, hazard ratio; IPSM, International Prognostic Scoring System for Mastocytosis; KPS, Karnofsky Performance Status; No, number of patients; OS, overall survival; PFS, progression-free survival; PR, partial remission; RRM, relapse-related mortality; TRM, transplant-related mortality

Limitations include the retrospective design, which precludes causal inference: our eligibility definition may not fully capture clinical transplant eligibility, which also depends on comorbidities, organ function, donor availability, and patient preference. Furthermore, TKI response is a post-treatment marker that may reflect favorable disease biology, so our data do not permit a direct comparison of TKI-directed and transplant-based strategies. Additional limitations are the heterogeneity in pre-transplant therapies, patient selection for allo-HCT, and transplant practices across centers and time (1998–2022), although center experience and year of diagnosis were included as covariates. Further constraints are the underrepresentation of avapritinib-treated patients, warranting cautious interpretation in current practice, and the limited registry granularity, which precluded attribution of post-transplant relapse to the mast cell or the associated myeloid compartment. Finally, the subtype-stratified analyses were exploratory and unadjusted for multiple testing.

Further investigations are needed to validate our findings in larger prospective cohorts and to address (1) the optimal time point of allo-HCT during KIT-directed treatment, including possible interactions between these treatments, (2) the prognostic significance of *KIT* D816V variant allele frequency and minimal residual disease status at transplant, and (3) the best conditioning regimen and graft-versus-host disease prophylaxis.

In conclusion, our results suggest that allo-HCT provides a meaningful survival benefit in SM-AML but not in other AdvSM subtypes, supporting a more selective role of allo-HCT in the modern therapeutic era. For patients with ASM, MCL, or SM-AHN without AML, our data support a central role for TKI-directed therapy, although these findings are hypothesis-generating and require prospective validation. IPSM at transplantation appears to be a practical and broadly applicable tool to inform transplant decision-making and timing.

## Supplementary Information

Additional file1 (DOCX 1946 KB)

## Data Availability

All data generated or analysed during this study are included in this published article and its supplementary information files.

## Abbreviations

AdvSM: Advanced systemic mastocytosis
AHN: Associated hematological neoplasm
Allo-HCT: Allogeneic hematopoietic cell transplantation
AML: Acute myeloid leukemia
ASM: Aggressive systemic mastocytosis
CI: Confidence interval
CR: Complete remission
ECNM: European competence network on mastocytosis
HR: Hazard ratio
IPSM: International prognostic scoring system for mastocytosis
KPS: Karnofsky performance status
MARS: Mutation-adjusted risk score
MCL: Mast cell leukemia
OS: Overall survival
PFS: Progression-free survival
PR: Partial remission
SM: Systemic mastocytosis
SM-AHN: Systemic mastocytosis with an associated hematological neoplasm
SM-AML: Systemic mastocytosis associated with an acute myeloid leukemia
RRM: Relapse-related mortality
TKI: Tyrosine Kinase Inhibitor
TRM: Transplant-related mortality
WHO: World Health Organization
