# Supplementary material for: Subtype-specific outcome after allogeneic hematopoietic cell transplantation in advanced systemic mastocytosis: a registry study of the European competence network on mastocytosis
Source: Exp Hematol Oncol. 2026 Aug 20;15:79. doi: 10.1186/s40164-026-00819-8 (PMC13491673; doi:10.1186/s40164-026-00819-8)
Supplement: Supplementary file 1 [file 40164_2026_819_MOESM1_ESM.docx]

**Supplementary Appendix**

**Subtype-Specific Outcome after Allogeneic Hematopoietic Cell Transplantation in Advanced Systemic Mastocytosis:**

**A Registry Study of the European Competence Network on Mastocytosis**

Stefan Haug^1^, Wolfgang R. Sperr^2,3^, Claudia Wehr^4^, Federico Bonofiglio^5^, Saskia K. Klein^6^, Hanneke C. Kluin-Nelemans^6^, André B. Mulder^7^, Olivier Hermine^8^, Julien Rossignol^8^, Felipe Suarez^8^, Ambroise Marcais^9^, William Shomali^10^, Cecelia Perkins^10^, Deborah Christen^11^, Werner Rabitsch^12^, Emir Hadzijusufovic^2,3^,‬ Jaroslava Voglová^13^, Madlen Jentzsch^14^, Juliana Schwaab^15^, Johannes Lübke^15^, Mattias Mattsson^16^, Hans Hagglund^16^, Ingunn Dybedal^17^, Daniel Baffoe^17^, Patrizia Bonnadonna^18^, Massimiliano Bonifacio^19^, Ilaria Tanasi^19^, Mariarita Sciumè^20^, Marek Niedoszytko^21^, Aleksandar Gorska^21^, Andrzej Mital^22^, Massimo Triggiani^23^, Roberta Parente^23^, Akif Selim Yavuz^24^, Chiara Elena^25^, Luca Malcovati^25^, Jacqueline Ferrari^25^, Friederike Wortmann^26^, Knut Brockow^27^, Tobias Michael Franz^27^, Michael Makris^28,29^, Sotirios G Papageorgiou^28,30^, Christine Breynaert^31^, Toon Leven^31^, Irena Angelova-Fischer^32^, Alex Stefan^33^, Judit Várkonyi^34^, Vito Sabato^35^, Tanja Daniela Schug^36^, Paul LA van Daele^37^, Anna Belloni Fortina^38^, Francesca Caroppo^39^, Theo Gulen^40^, Marc Heizmann^41^, Karin Hartmann^42,43,44^, Axel Rüfer^45^, Michael Doubek^46^, Jens Panse^11^, Jason Gotlib^10^, Michel Arock^47^, Hanneke N.G. Oude Elberink^48^, Andreas Reiter^15^, Peter Valent^2,3^, Khalid Shoumariyeh^4,49^

^1^Institute of Epidemiology and Prevention, Faculty of Medicine and Medical Center - University of Freiburg, Freiburg, Germany;

^2^Department of Internal Medicine I, Division of Hematology and Hemostaseology, Medical University of Vienna, Vienna, Austria;

^3^Ludwig Boltzmann Institute for Hematology and Oncology, Medical University of Vienna, Vienna, Austria;

^4^Department of Medicine I, Medical Center-University of Freiburg, Faculty of Medicine, University of Freiburg, Freiburg, Germany;

^5^National Research Council of Italy (CNR), Lerici, Italy;

^6^University Medical Center Groningen, Department of Hematology, University of Groningen, Groningen, The Netherlands;

^7^University Medical Center Groningen, Department of Laboratory Medicine, University of Groningen, Groningen, The Netherlands;

^8^Hôpital Necker-Enfants Malades, APHP, Imagine Institute INSERM U1163, Université Paris Cité, Centre national de référence des mastocytoses, Paris, France;

^9^Service Hématologie, Hôpitaux universitaires de Strasbourg, Strasbourg, France;

^10^Stanford Cancer Institute/Stanford University School of Medicine, Stanford, CA, USA;

^11^Department of Oncology, Haematology, Haemostaseology and Stem Cell Transplantation, University Hospital RWTH Aachen, Aachen, Germany and Center for Integrated Oncology Aachen Bonn Cologne Düsseldorf (CIO ABCD), Aachen, Germany;

^12^Department of Internal Medicine I, Bone Marrow Transplantation Unit, Medical University of Vienna, Vienna, Austria;

^13^4th Department of Internal Medicine and Hematology, University Hospital Hradec Králové and Charles University, Hradec Králové, Czech Republic;

^14^Medizinische Klinik I – Hämatologie, Zelltherapie, Hämostaseologie und Infektiologie, Universitätsklinikum Leipzig AöR, Leipzig, Germany;

^15^Department of Hematology and Oncology, University Hospital Mannheim, Heidelberg University, Mannheim, Germany;

^16^Department of Medical Sciences, Hematology, Uppsala University, Uppsala, Sweden;

^17^Departments of Hematology and Pharmacology, Oslo University Hospital, Rikshospitalet, Oslo, Norway;

^18^Allergy Unit, Department of Medicine, Azienda Ospedaliera Universitaria Integrata di Verona, Verona, Italy;

^19^Department of Engineering for Innovation Medicine, Section of Innovation Biomedicine, Hematology Area, University of Verona, Verona, Italy;

^20^S.C. Ematologia, Fondazione IRCCS Ca' Granda Ospedale Maggiore Policlinico, Milan, Italy;

^21^Department of Allergology, Medical University of Gdansk, Gdansk, Poland;

^22^Department of Haematology and Transplantology, Medical University of Gdansk, Gdansk, Poland;

^23^Division of Allergy and Clinical Immunology, University of Salerno, Salerno, Italy;

^24^Division of Hematology, Istanbul Medical School, University of Istanbul, Istanbul, Turkey;

^25^Division of Molecular Hematology and Precision Medicine, Fondazione IRCCS Policlinico San Matteo, Pavia, Italy;

^26^Department of Hematology and Oncology, University Hospital of Schleswig-Holstein (UKSH) and University Cancer Center of Schleswig Holstein (UCCSH), Lübeck, Germany;

^27^Department of Dermatology and Allergy Biederstein, TUM School of Medicine and Health, Technical University of Munich, Munich, Germany;

^28^National Center of Expertise for Mastocytosis and Mast Cell Disorders, 2nd Dpt. of Dermatology and Venereology, National and Kapodistrian University of Athens, University General Hospital ‘Attikon’, Athens, Greece;

^29^Allergy Unit, 2nd Dpt. of Dermatology and Venereology, National and Kapodistrian University of Athens, University General Hospital ‘Attikon’, Athens, Greece;

^30^2nd Department of Internal Medicine, Propaedeutic and Research Institute, Hematology Unit, University of Athens, Attikon University Hospital, Athens, Greece;

^31^KU Leuven Department of Microbiology, Immunology and Transplantation, Allergy and Clinical Immunology Research Group and MASTeL, UZ Leuven, Leuven, Belgium;

^32^Department of Dermatology, Kepler University Hospital, Linz, Austria;

^33^Department of Internal Medicine, Kepler University Hospital, Linz, Austria;

^34^Department of Hematology, Semmelweis University, Budapest, Hungary;

^35^Universiteit Antwerpen, Campus Drie Eiken, Antwerp, Belgium;

^36^Univ. Klinik für Dermatologie, Medical University of Graz, Graz, Austria;

^37^Department of Internal Medicine and Department of Immunology, Erasmus University Rotterdam, Rotterdam, The Netherlands;

^38^Pediatric Dermatology Unit, Department of Women’s and Children’s Health (SDB), University of Padova, Padova, Italy;

^39^Pediatric Dermatology Unit, Department of Medicine (DIMED) & Department of Women’s and Children’s Health (SDB), University of Padova, Padova, Italy;

^40^Department of Respiratory Medicine and Allergy; Karolinska University Hospital Huddinge, Stockholm, and Department of Medicine Solna, Karolinska Institutet, Stockholm, Sweden;

^41^Division of Oncology, Haematology and Transfusion Medicine, Kantonsspital Aarau, Aarau, Switzerland;

^42^Division of Allergy, Department of Dermatology, University Hospital Basel and University of Basel, Basel, Switzerland;

^43^Department of Clinical Research, University Hospital Basel and University of Basel, Basel, Switzerland;

^44^Department of Biomedicine, University Hospital Basel and University of Basel, Basel, Switzerland;

^45^Department of Hematology, Luzerner Kantonsspital, University of Lucerne, Lucerne, Switzerland;

^46^University Hospital and Masaryk University, Brno, Czechia;

^47^Pitié-Salpêtrière Hospital, University of Sorbonne Paris Cité, APHP Centre national de référence des mastocytoses, Paris, France;

^48^Department of Allergology, University Medical Center Groningen, University of Groningen, and Groningen Research Institute for Asthma and COPD, Groningen, The Netherlands;

^49^German Cancer Consortium (DKTK), Partner Site Freiburg and German Cancer Research Center (DKFZ), Heidelberg, Germany.

# Supplementary Methods

## Study Design and Patient Population

This retrospective cohort study analyzed data from the European Competence Network on Mastocytosis (ECNM) registry, established in 2012. Details on study design, eligibility criteria, disease categories, ethics approval, informed consent, and prognostic variables have been published previously (1). The eighth data wave (extracted November 2023) contained clinical, laboratory, and pathologic information on 5,465 patients with mastocytosis from 31 European centers (15 countries) and one US center (Stanford Cancer Institute, CA). AdvSM diagnoses were established according to WHO and consensus group criteria and ranged from April 14, 1988 to December 6, 2022 (2-7).

The study adhered to the Declaration of Helsinki and was approved by participating centers' institutional Ethics Committees. All patients provided written informed consent.

## Detailed Patient Selection

For comparative analyses of allo-HCT and tyrosine kinase inhibitor (TKI) treatment effects, we applied a complete-case approach restricted to patients theoretically eligible for allo-HCT. From the primary cohort of 631 AdvSM patients, we excluded patients with: WHO performance status >3, age <18 or >75 years at AdvSM diagnosis, or missing data for key prognostic factors. The final complete-case cohort comprised 419 patients (66% of the primary cohort), including 63 patients who underwent allo-HCT, and was used for all adjusted survival analyses from AdvSM diagnosis (**Supplementary Figure 3**).

Post-transplant outcome analyses included all 69 allo-HCT recipients. Given limited sample size, the primary multivariate model was restricted to three clinically essential covariates (Karnofsky Performance Score [KPS], IPSM risk group, remission status), with complete data available for 52 patients (overall survival [OS] analysis) and 50 patients (progression-free survival [PFS] analysis).

## Missing Data Assessment

Missing data patterns were evaluated by comparing baseline characteristics between patients included in the complete-case cohort (*n = 419*) and those excluded solely due to missing data (*n = 122*), excluding patients removed for age or performance status criteria. Comparisons used Wilcoxon rank-sum tests for continuous variables and Fisher's exact test for categorical variables (**Supplementary Table 1**).

Patients excluded due to missing data were more likely to be male (70% vs 59%, *p*=0.034) and less likely to have documented TKI treatment (40% vs 54%, *p*=0.003) or allo-HCT (2% vs 16%, *p*<0.001). LDH at AdvSM diagnosis was higher in excluded patients (median 215 U/L vs 172 U/L, *p*=0.003), although both values remained within the normal range (<250 U/L). Complete-case analysis provides valid estimates when data are missing at random (MAR), that is, when missingness depends only on observed variables rather than unobserved outcomes. We included sex in our models as a covariate. Under the MAR assumption, controlling for sex should reduce potential bias, although we cannot definitively rule out that unmeasured factors influenced missingness. Given that all key prognostic factors associated with missingness were included in multivariate models, we considered the MAR assumption plausible.

## Treatment Exposures

Allo-HCT was performed according to institutional protocols, with details on conditioning intensity, donor source, and graft-versus-host disease prophylaxis recorded per center standards.

TKI therapy included any KIT-targeting agent (midostaurin, avapritinib, imatinib, or investigational TKIs). Patients were categorized as responders (complete response, partial response, or clinical improvement) versus non-responders (stable disease or progressive disease) based on pure pathologic response reported by the centers.

## AdvSM Prognostic Scores

The International Prognostic Scoring System for Mastocytosis (IPSM), developed within the ECNM cohort, stratifies patients into risk groups 1-4 based on six variables: age ≥60 years, tryptase ≥125 ng/mL, leukocytes ≥16×10⁹/L, hemoglobin ≤11 g/dL, platelets ≤100×10⁹/L, and skin involvement IPSM was calculated at AdvSM diagnosis and, for transplanted patients, at allo-HCT prior to conditioning (8).

The Mutation-Adjusted Risk Score (MARS) stratifies patients into low, medium, and high-risk groups based on age >60 years, hemoglobin <10 g/dL, platelets <100×10⁹/L, and presence of *SRSF2*/*ASXL1*/*RUNX1* (S/A/R) mutations (9). MARS was calculated at allo-HCT prior to conditioning.

## Study Endpoints

OS was defined as time from AdvSM diagnosis (or from allo-HCT for transplant analyses) to death from any cause or last follow-up. PFS after allo-HCT was defined as time from allo-HCT to disease progression, relapse, death, or last follow-up. Cause-specific mortality in the allo-HCT cohort was classified as: (1) relapse-related mortality (RRM), (2) transplant-related mortality (TRM), or (3) other causes.

Administrative censoring was applied at 60 months from AdvSM diagnosis (primary analyses) or from allo-HCT (transplant analyses), as patient numbers at risk declined substantially beyond these timepoints.

## Statistical Analyses

Continuous variables were summarized by median and interquartile range; categorical variables by proportion. OS and PFS probabilities were estimated using Kaplan-Meier methods with log-rank tests for group comparisons.

Univariate and multivariate Cox proportional hazards regression models assessed prognostic factors and treatment associations with OS and PFS. Covariates were selected a priori based on clinical relevance and established prognostic significance. Results are presented as hazard ratios (HRs) with two-sided 95% confidence intervals (CIs). P-values <0.05 were considered statistically significant. Proportional hazards assumptions were evaluated using Schoenfeld residuals.

### Complete-Case Cohort Models (n = 419)

For the complete-case cohort, models included time-dependent covariates and time-fixed covariates as follows.

*Time-dependent covariates:*

- Allo-HCT treatment: Coded as 1 from date of transplantation, 0 otherwise
- TKI response status: Coded as 1 from TKI initiation date for patients achieving response (complete response, partial response, or clinical improvement), 0 otherwise

*Time-fixed covariates:*

- Sex: Male vs female (reference)
- AdvSM subtype: SM-AML, ASM±AHN, MCL±AHN, each compared to other SM-AHN (reference)
- KIT D816V mutation status: Positive vs negative/not tested (reference)
- WHO performance status: 0-3, modeled per 1-point increase
- IPSM risk group: 1-4, modeled per 1-category increase
- Center experience: Number of AdvSM patients treated at center, modeled per 10-patient increase
- Year of diagnosis: Modeled per decade from first AdvSM diagnosis in study population

Multivariate models included all pre-specified covariates simultaneously to assess independent prognostic effects. For continuous variables modeled per unit increase, the HR represents the change in hazard per specified increment.

Univariate analyses employed Cox proportional hazards regression models to assess prognostic factors and treatment associations with OS individually (**Supplementary Tables 2** **and 3**). Additionally, Kaplan-Meier survival estimates with log-rank tests compared OS across categorical groups for all variables included in multivariate models (**Supplementary Figure 1**).

### TKI Response Timing Considerations

Because exact TKI response dates were incompletely documented in the registry, responders were coded from treatment initiation rather than response date. This approach assumes minimal lag between treatment start and response (<6 months), which is clinically reasonable, as KIT inhibitor responses occur within the first months of treatment when effective, and non-responders typically discontinue therapy within this timeframe.

To assess potential immortal-time bias (wherein patients must survive to response assessment to be classified as responders), we performed sensitivity analyses assuming all responses occurred 3 or 6 months after TKI initiation. Within this clinically plausible response window, hazard ratios remained identical to the primary analysis (HR 0.41 for both scenarios), confirming that results are robust to response timing assumptions and that guarantee-time bias is negligible.

### TKI Treatment Sensitivity Analysis

As a sensitivity analysis, TKI treatment initiation (regardless of response) was modeled as a time-dependent covariate (coded as 1 from TKI start date, 0 otherwise) instead of TKI response status (**Supplementary Figure 2**).

### Subtype-Stratified Analyses

Separate univariate and multivariate Cox models were fitted within each AdvSM subtype category (SM-AML, ASM±AHN, MCL±AHN, other SM-AHN) using identical covariates to enable comparison of prognostic factor effects across disease subtypes. Given the exploratory nature of these analyses and limited sample sizes in some strata, no adjustment for multiple testing was applied, and results should be interpreted as hypothesis-generating.

### Allo-HCT Subcohort Models (n = 69)

To ensure model stability given limited events (*n = 24* for OS, *n = 28* for PFS), the primary multivariate model included three covariates selected a priori based on clinical relevance for transplant outcomes:

- KPS: Dichotomized as ≥90 vs <90 (reference)
- IPSM risk group at allo-HCT: 1-4, modeled per 1-category increase
- Remission status before allo-HCT: Complete response or partial response vs stable disease or progressive disease (reference)

An extended multivariate model (sensitivity analysis) additionally examined: AdvSM subtype, sex, conditioning regimen intensity (myeloablative vs reduced intensity), KIT D816V mutation status, and prior treatment response including prior TKI response (**Supplementary Table 4**).

Univariate analyses employed Cox proportional hazards regression models to assess prognostic factors and treatment associations with OS and PFS (**Supplementary Tables 4**).

### Competing Risk Analyses

Cumulative incidence functions estimated RRM, TRM, and other causes of death, treating each as competing events. Cumulative incidence curves were stratified by IPSM risk group at allo-HCT. Gray's test compared cumulative incidence across risk groups.

## Software

All statistical analyses were performed using R version 4.1.0 (R Foundation for Statistical Computing, Vienna, Austria).

# Supplementary Results

## Patient characteristics and treatment patterns of the AdvSM cohort

From the ECNM registry (*n = 5,465*), we excluded patients with cutaneous mastocytosis or mastocytosis in the skin (*n = 2,068*), ISM, BMM, or SSM (*n = 2,697*), MCS (*n = 4*), or insufficient follow-up (FU) (*n = 65*), yielding a primary cohort of 631 patients with AdvSM (**Supplementary Figure 3**). Of these, 566 (90%) had AdvSM at initial diagnosis and 65 (10%) progressed to AdvSM during FU. Sixty-nine patients (11%) underwent allo-HCT at least once (**Supplementary Figure 3** and **Supplementary Table 6**).

Median age at AdvSM diagnosis was 64 years (IQR 54-71), and 394 patients (62%) were male. Among SM-AHN patients, the most common associated hematological neoplasms were MDS/MPN overlap syndromes (*n = 154*, 37%), MPN (*n = 112*, 27%), MDS (*n = 62*, 15%), and AML (*n = 41*, 10%). *KIT* D816V was detected in 86% of the patients. IPSM risk groups among 589 evaluable patients were: group 1 (*n = 110*, 19%), group 2 (*n = 115*, 20%), group 3 (*n = 260*, 46%), and group 4 (*n = 84*, 15%) (**Supplementary Table 6**). Detailed baseline patient characteristics of the AdvSM cohort are shown in **Supplementary Table 6**.

Detailed information about TKI treatment in the AdvSM cohort as well as in the allo-HCT cohort are provided in the section “TKI treatment characteristics of the AdvSM and allo-HCT cohort” below.

## Overall Survival in AdvSM

At a median follow-up of 31.2 months (IQR 12.2-58.5), 292 deaths occurred in the primary cohort (*n = 631*). Median OS was 48.0 months (95% CI 43.1-55.6), with 2- and 5-year OS probabilities of 71% (95% CI 67-75%) and 43% (95% CI 39-48%), respectively (**Supplementary Figure 4A**).

OS differed significantly by AdvSM subtype (log-rank *p*<0.001, **Supplementary Figure 5A**). Five-year OS was highest for other SM-AHN (70%, 95% CI 62-79%), followed by ASM±AHN (36%, 95% CI 31-43%), MCL±AHN (31%, 95% CI 21-44%), and SM-AML (29%, 95% CI 16-54%). Similarly, OS was strongly associated with IPSM risk group (log-rank *p*<0.001; **Supplementary Figure 5B**). Five-year OS decreased progressively from IPSM risk group 1 (87%, 95% CI 80-95%) to risk group 4 (12%, 95% CI 6-24%).

## Prognostic factors influencing overall survival in AdvSM

To assess the potential benefit of allo-HCT for AdvSM patients while accounting for potential confounders, we defined an allo-HCT-eligible (age 18–75 years, WHO performance status ≤3) complete-case cohort of 419 patients, including 63 transplanted patients (**Supplementary Figure 3, Supplementary Table 3**).

Patients who underwent allo-HCT differed significantly from those who did not in several baseline characteristics. Those who received allo-HCT were younger at diagnosis (median age 54 vs. 63.5 years, *p*<0.001), more frequently had SM-AML (29% vs. 3%, *p*<0.001) and abnormal karyotypes (39% vs. 12%, *p*<0.001), while ASM +/- AHN was less frequent (33% vs 57%, *p*<0.001).Transplanted patients furthermore presented with lower serum tryptase, hemoglobin, and platelet levels but higher LDH at diagnosis (**Supplementary Table 7**).

In a multivariate analysis, IPSM risk group emerged as the strongest predictor of mortality (HR per 1-category increase 2.21, 95% CI 1.86-2.64; *p*<0.001; **Supplementary Figure 5C, Supplementary Table 2**). Additional adverse prognostic factors included poor WHO performance status (HR per 1-point increase 1.46, 95% CI 1.20-1.77; *p*<0.001), male sex (HR 1.37, 95% CI 1.03-1.82; *p*=0.031), and AdvSM subtype. MCL±AHN (HR 2.08, 95% CI 1.27-3.40; *p*=0.003), SM-AML (HR 1.93, 95% CI 1.05-3.56; *p*=0.035), and ASM±AHN (HR 1.75, 95% CI 1.14-2.67; *p*=0.010) were associated with a significantly higher mortality risk compared with other SM-AHN. *KIT* D816V positivity was associated with improved OS (HR 0.67, 95% CI 0.46-0.96; *p*=0.028).

Importantly, allo-HCT was not associated with improved OS in the complete-case cohort (HR 1.03; 95% CI 0.64-1.66; *p*=0.90), whereas TKI response was strongly associated with improved survival (HR 0.41; 95% CI 0.28-0.59; *p*<0.001) (**Supplementary Figure 5C, Supplementary Table 2**). Treatment at higher-volume centers was associated with a modestly better OS (HR per 10 additional patients 0.94, 95% CI 0.89-0.99; *p*=0.030), while a later year of AdvSM diagnosis showed a trend towards worse survival (HR per decade 1.25, 95% CI 0.93-1.66; *p*=0.14).

## Subtype-stratified analyses of prognostic factors for overall survival

In subtype-stratified multivariate Cox regression models using the complete-case cohort (*n = 419*), IPSM risk group retained prognostic significance across all subtypes except SM-AML (HR per category: ASM±AHN 2.42, 95% CI 1.93-3.05, *p*<0·001; MCL+/-AHN 2.00, 95% CI 1.31-3.05, *p*=0.001; other SM-AHN 2.07, 95% CI 1.33-3.23, *p*=0·001; SM-AML 1.57, 95% CI 0.81-3.03, *p*=0.18) (**Figure 1, Supplementary Table 3**).

Similarly, a poor WHO performance status predicted worse survival across subtypes, significantly in ASM±AHN (HR 1.54, 95% CI 1.18-2.00, *p*=0.002) and except for SM-AML (HR 0.72, 95% CI 0.30-1.77, *p*=0.48).

Male sex was associated with significantly adverse outcome in ASM±AHN (HR 1.77, 95% CI 1.20-2.61, *p*=0.004), while *KIT* D816V positivity was associated with improved survival only in MCL±AHN (HR 0.46, 95% CI 0.24-0.88, *p*=0.018).

Most notably, the analysis suggests that allo-HCT has a survival benefit exclusively in SM-AML (HR 0.20, 95% CI 0.05-0-78; *p*=0.020), with no survival benefit in other subtypes. TKI response was significantly associated with improved survival in ASM±AHN (HR 0.29, 95% CI 0.18-0.46; *p*<0.001) and MCL±AHN (HR 0.27, 95% CI 0.08-0.91; *p*=0.035).

Treatment at higher-volume centers was associated with better survival only in ASM±AHN (HR per 10 additional patients 0.91, 95% CI 0.85-0.98; *p*=0.012), while year of diagnosis showed divergent subtype-specific effects: later diagnosis was associated with worse survival in ASM±AHN (HR per decade 1.55, 95% CI 1.03-2.33; *p*=0.034) but better survival in SM-AML (HR per decade 0.34, 95% CI 0.13-0.92; *p*=0.034).

## Patient characteristics and survival outcomes after allo-HCT cohort

Among 69 transplanted patients, median age at allo-HCT was 53 years (IQR 46-60 years), with a median time from AdvSM diagnosis to allo-HCT of 9.4 months (IQR 4.8-20.9 months). Detailed patient characteristics of the allo-HCT cohort and transplant modalities are detailed in **Supplementary Table 8**.

Median post-transplant PFS was 23.5 months (95% CI 10.8->60.0), with 2- and 5-year PFS probabilities of 50% (95% CI 39-64%) and 36% (95% CI 25-53%), respectively (**Supplementary Figure 6A**). TRM occurred in 36% (12/69) of the cases.

At a median post-transplant FU of 26.2 months (IQR 8.3-51.7), median OS was 49.6 months (95% CI 28.2>60.0), with 2- and 5-year OS probabilities of 63% (95% CI 53-76%) and 46% (95% CI 34-61%), respectively (**Supplementary Figure 6B**).

## Prognostic factors influencing outcome after allo-HCT

We assessed prognostic factors for OS and PFS after allo-HCT using multivariate Cox regression. To ensure model stability given the limited sample size, we focused on three clinically relevant covariates: IPSM risk group at transplantation, KPS, and remission status prior to conditioning. In multivariate analysis (*n = 52* for OS and *n = 50* for PFS), higher IPSM risk group (HR per category 1.68, 95% CI 1.07-2.65; *p*=0.026) was an independent predictor of worse OS, while KPS ≥90 was associated with improved OS (HR 0.37, 95% CI 0.16-0.85; *p*=0.020). For PFS, higher IPSM risk group was the only independent predictor (HR per category 1.99, 95% CI 1.27-3.11; *p*=0.003) (**Figure 2A, Supplementary Table 4**). Achievement of complete or partial remission before transplantation showed a non-significant trend toward improved outcomes.

The prognostic impact of the IPSM risk group is illustrated with stratified Kaplan-Meier estimates in **Figure 2B** and **2C** and **Supplementary Figure 7A and 7B**. When stratified into favorable (IPSM groups 1-2) versus unfavorable (groups 3-4) risk categories, 2-year OS was 72% (95% CI 58-90%) versus 48% (95% CI 34-70%), and 2-year PFS was 60% (95% CI 45-81%) versus 32% (95% CI 19-54%), respectively (log-rank *p*<0.01 for both comparisons). Outcomes were particularly poor in IPSM risk group 4, where all 7 patients experienced disease relapse/progression within 12 months and died within 36 months after allo-HCT.

In a multivariate model including KPS, remission status, and MARS at allo-HCT, high-risk MARS showed a trend toward worse OS (HR 1.50, 95% CI 0.89-2.53; *p*=0.13) and PFS (HR 1.64, 95% CI 0.98-2.73; *p*=0.058) but did not reach statistical significance in this cohort (**Supplementary Table 5**).

## Causes of death after allo-HCT

Among 33 deaths after allo-HCT, 16 (48%) were attributed to disease relapse or progression (RRM), 12 (36%) to TRM, and 5 (15%) to other causes. Two-year cumulative incidence was 18% (95% CI 10-28%) for RRM, 16% (95% CI 9-26%) for TRM, and 3% (95% CI 1-9%) for other causes, with 5-year estimates of 26% (95% CI 15-37%), 18% (95% CI 10-29%), and 10% (95% CI 4-21%), respectively (**Supplementary Table 9**).

Both RRM and TRM increased with higher IPSM risk category at allo-HCT (**Figure 2D** and **Supplementary Table 10**). The primary causes of TRM were graft-versus-host disease (GVHD) in 7 patients (54%), infection in 5 patients (38%), and thrombocytopenia-associated hemorrhagic shock in one patient (8%).

## Sensitivity analysis TKI treatment vs. TKI response

A sensitivity analysis demonstrated that TKI treatment without documented response was not associated with improved survival (HR 0.88, 95% CI 0.65-1.17; *p*=0.37; **Supplementary Figure 2**), indicating that achieving response, rather than treatment itself, confers survival benefit.

## Multivariate analysis of individual IPSM components

Analysis of individual IPSM components in the allo-HCT cohort demonstrated that age ≥60 years, tryptase ≥125 ng/mL, and WBC ≥16×10⁹/L remained independently prognostic for OS and PFS after transplantation (**Supplementary Table 11**).

## TKI treatment characteristics of the AdvSM and allo-HCT cohort

Overall, 319 of 631 patients (51%) in the primary AdvSM cohort were documented to have received TKI treatment, with the individual substances listed in **Supplementary Table 12**. Among TKI-treated patients, midostaurin was used most frequently (*n = 275*, 86%), followed by imatinib (*n = 52*, 16%) and avapritinib (*n = 18*, 6%). In many cases, different TKIs were administered sequentially: midostaurin treatment often followed an initial imatinib therapy, whereas avapritinib tended to be used after midostaurin. This sequence reflects the chronological introduction of the respective substances into clinical practice (**Supplementary Table 12**). Of all 319 TKI-treated patients, 163 (51%) had a documented response to therapy, recorded in 14 of 37 (38%) transplanted and 149 of 282 (53%) non-transplanted patients. Although the proportion of non-responders was higher in transplanted patients (62% vs. 47%), this difference was not statistically significant (*p*=0.12, Fisher's exact test).

Of the 69 transplanted patients, 34 (49%) underwent allo-HCT before and 35 (51%) after EMA approval of midostaurin (September 2017). A total of 37 transplanted patients (54%) received TKI treatment. As in the full AdvSM cohort, midostaurin was by far the most common agent, administered to 32 patients (87%). TKI treatment was given predominantly before allo-HCT (31 patients), while 7 patients received TKIs after transplantation, including one patient treated both before and after. Notably, no patient received avapritinib prior to allo-HCT, consistent with the more recent introduction of this agent. Details for all TKIs administered in the allo-HCT cohort are provided in **Supplementary Tables 13A and 13B**.

## Characteristics and outcomes of patients with SM-AML

The complete-case cohort included 30 patients with SM-AML, of whom 18 underwent allo-HCT and 12 did not. Baseline characteristics, treatment details, and outcomes for both groups are provided in **Supplementary Table 14**.

Transplanted patients were younger at diagnosis (median 59 vs 64.5 years), more frequently KIT D816V positive (94% vs 67%), and more frequently S/A/R positive (64% vs 0%, although S/A/R status was available in only three non-transplanted patients). They also presented with lower platelet counts (median 54.5 vs 81 G/L) and higher LDH (median 265.5 vs 181.5 U/L) at diagnosis, whereas IPSM risk group and WHO performance status were comparable between groups. TKI treatment was documented more frequently in transplanted patients (56% vs 25%).

Among transplanted patients, the median time from AdvSM diagnosis to allo-HCT was 4.1 months (IQR 3.4-6.1), 56% were in complete or partial remission before transplantation, and 65% received myeloablative conditioning. Post-transplant overall survival was 83% (95% CI 68-100) at 1 year and 53% (95% CI 30-95) at 5 years, with corresponding progression-free survival of 83% (95% CI 68-100) and 45% (95% CI 24-84). Relapse occurred in 8 of 18 patients (44%); among the 6 deaths, 2 (33%) were relapse-related, 3 (50%) transplant-related, and 1 (17%) due to other causes.

These data indicate that transplanted SM-AML patients represent a selected population, exhibiting features of both favorable selection (younger age, more frequent cytoreductive treatment) and more aggressive disease biology (higher LDH, lower platelets, more frequent S/A/R mutations). Given the small number of patients, particularly in the non-transplanted group, these findings should be interpreted as exploratory.

# Supplementary Tables

## **Supplementary Table 1:** Comparison of baseline characteristics between the complete-case cohort and patients excluded due to missing data.

|  | **AdvSM complete cases** | **AdvSM missing data** | **P value** |
| --- | --- | --- | --- |
|  | *N* = 419 | *N* = 122 |  |
| **Sex (male)**, %, (N) | 59 (249/419) | 70 (86/122) | **3.38E-02** |
| **Age**, years, median (IQR, N) | 62 (52-68, 419) | 62.5 (51-69, 122) | 9.20E-01 |
| **WHO Performance Status**, median (IQR, N) | 1 (1-2, 419) | 1 (0-1, 84) | 6.55E-02 |
| **Follow up (months),** median (IQR, N) | 35.7 (15.57-64.48, 419) | 27.1 (11.93-57.91, 122) | 1.16E-01 |
| **AdvSM subtypes** |  | | |
| - SM-AML % (N) | 7 (30/419) | 4 (5/122) | 2.97E-01 |
| - MCL±AHN % (N) | 14 (59/419) | 7 (9/122) | 6.15E-02 |
| - ASM +/- AHN | 53 (223/419) | 55 (67/122) | 7.58E-01 |
| - Other SM-AHN % (N) | 26 (107/419) | 34 (41/122) | 8.42E-02 |
| **IPSM Score,** median (IQR, N) | 3 (2-3, 419) | 3 (2-3, 67) | 7.77E-01 |
| - Risk group 1 % (N) | 22 (92/419) | 22 (15/67) | 1.00E+00 |
| - Risk group 2 % (N) | 23 (95/419) | 19 (13/67) | 6.36E-01 |
| - Risk group 3 % (N) | 41 (173/419) | 51 (34/67) | 1.83E-01 |
| - Risk group 4 N % (N) | 14 (59/419) | 7 (5/67) | 1.73E-01 |
| ***KIT* D816V positive,** % (N) | 85 (357/419) | 85 (72/85) | 8.68E-01 |
| **Aberrant Karyotype,** % (N) | 18 (46/252) | 25 (11/44) | 3.03E-01 |
| **S/A/R status positive,** % (N) | 44 (55/126) | 25 (5/20) | 1.45E-01 |
| **Lab values at AdvSM diagnosis** |  | | |
| - Serum Tryptase, µg/L, median (IQR, N) | 147 (55.85-268.5, 419) | 122 (34.3-250.75, 82) | 2.01E-01 |
| - WBC, G/L, median (IQR, N) | 7.8 (4.6-12.9, 419) | 8.5 (4.93-16.28, 110) | 3.11E-01 |
| - Eosinophils, G/L, median (IQR, N) | 0.18 (0.04-0.71, 382) | 0.27 (0.02-0.69, 89) | 7.24E-01 |
| - Hemoglobin, g/dL, median (IQR, N) | 11 (9.3-12.9, 419) | 11.2 (9.3-13.3, 107) | 6.85E-01 |
| - Platelets, G/L, median (IQR, N) | 158 (80-264, 419) | 153 (81-246.5, 107) | 5.58E-01 |
| - LDH, U/L, median (IQR, N) | 172 (136-234, 381) | 215 (154-286.5, 75) | **3.42E-03** |
| - AP, U/L, median (IQR, N) | 136.5 (83.87-274.75, 386) | 127 (79.75-272.75, 86) | 5.48E-01 |
| **Cytoreductive treatment** |  | | |
| - TKI, % (N) | 54 (227/419) | 40 (49/122) | **7.40E-03** |
| - Allo-HCT, % (N) | 16 (66/419) | 2 (3/122) | **2.07E-05** |
| - Polychemotherapy, % (N) | 15 (62/419) | 13 (16/122) | 7.70E-01 |
| - 2-CdA, % (N) | 27 (115/419) | 22 (27/122) | 2.93E-01 |
| - IFN-α, % (N) | 16 (65/419) | 16 (19/122) | 1.00E+00 |
| - Hydroxyurea, % (N) | 17 (70/419) | 17 (21/122) | 8.91E-01 |
| - NA, % (N) | 18 (77/419) | 29 (35/122) | **1.59E-02** |
| Continuous variables compared using Wilcoxon rank-sum test; categorical variables compared using Fisher's exact test.  Abbreviations: 2-CdA, Cladribine; AdvSM, advanced systemic mastocytosis; AHN, associated hematologic neoplasm; allo-HCT, allogeneic hematopoietic cell transplantation; AP, alkaline phosphatase; ASM, aggressive systemic mastocytosis; IFN-α, interferon alpha; IQR, interquartile range; IPSM, international prognostic scoring system in mastocytosis; LDH, lactate dehydrogenase; m, male; MCL, mast cell leukemia; n, number; NA, not available; S/A/R, *SRSF2*/*ASXL1*/*RUNX1*; SM-AHN, systemic mastocytosis with an associated hematologic neoplasm; SM-AML, systemic mastocytosis with an associated acute myeloid leukemia; TKI, tyrosine kinase inhibitor; WBC, white blood cells; WHO, world health organization. | | | |

## **Supplementary Table 2: Univariate and multivariate Cox proportional hazards regression analysis of prognostic factors for OS in the complete-case AdvSM cohort of patients from AdvSM diagnosis**.

|  | **Univariate** | | **Multivariate** | |
| --- | --- | --- | --- | --- |
| **Covariates** | **HR (95% CI)** | **P value** | **HR (95% CI)** | **P value** |
| **Time-dependent covariates** |  |  |  |  |
| Allo-HCT | 1.31 (0.88-1.95) | 0.185 | 1.03 (0.64-1.66) | 0.900 |
| TKI response | 0.64 (0.46-0.91) | **0.013** | 0.41 (0.28-0.59) | **<0.001** |
| **Time-fixed covariates** |  |  |  |  |
| *KIT* D816V positive | 0.72 (0.51-1.00) | 0.052 | 0.67 (0.46-0.96) | **0.028** |
| WHO Performance Status | 1.76 (1.50-2.07) | **<0.001** | 1.46 (1.20-1.77) | **<0.001** |
| Male sex | 1.65 (1.25-2.17) | **<0.001** | 1.37 (1.03-1.82) | **0.031** |
| IPSM | 2.39 (2.04-2.79) | **<0.001** | 2.21 (1.86-2.64) | **<0.001** |
| AdvSM subtype |  |  |  |  |
| Other SM-AHN | 1 |  | 1 |  |
| ASM +/- AHN | 2.37 (1.60-3.49) | **<0.001** | 1.75 (1.14-2.67) | **0.010** |
| MCL +/- AHN | 3.47 (2.20-5.49) | **<0.001** | 2.08 (1.27-3.40) | **0.003** |
| SM-AML | 3.26 (1.89-5.63) | **<0.001** | 1.93 (1.05-3.56) | **0.035** |
| N AdvSM/center | 1.08 (1.03-1.13) | **0.003** | 0.94 (0.89-0.99) | **0.030** |
| Year of diagnosis | 0.99 (0.76-1.28) | 0.914 | 1.25 (0.93-1.66) | 0.137 |
| Abbreviations: AdvSM, advanced systemic mastocytosis; AHN, associated hematologic neoplasm; allo-HCT, allogeneic hematopoietic cell transplantation; ASM, aggressive systemic mastocytosis; CI, confidence interval; HR, hazard ratio; IPSM, International Prognostic Scoring System for Mastocytosis; MCL, mast cell leukemia; N, number; SM, systemic mastocytosis; SM-AHN, systemic mastocytosis with an associated hematologic neoplasm; SM-AML, systemic mastocytosis with an associated acute myeloid leukemia; TKI, tyrosine kinase inhibitor; WHO, World Health Organization. | | | | |

Univariate and multivariate Cox proportional hazards regression analysis of prognostic factors for overall survival in the complete-case AdvSM cohort (*n* = 419, *n* deaths = 174). Allo-HCT treatment (coded as 1 from time of transplantation, 0 otherwise) and TKI response status (coded as 1 from initiation date of first TKI therapy achieving a response, 0 otherwise) were modeled as time-dependent covariates. All other covariates were time-fixed and assessed at time of AdvSM diagnosis. The multivariate model included all pre-specified covariates simultaneously. Hazard ratios (HR) >1 indicate increased risk of death; HR <1 indicate reduced risk. For variables modeled per unit increase (WHO performance status per 1-point increase, IPSM risk group per 1-category increase, center experience per 10-patient increase, year of diagnosis per decade), the HR represents the change in hazard per specified increment. *P*-values <0.05 were considered statistically significant.

|  | | Univariate | | Multivariate | |
| --- | --- | --- | --- | --- | --- |
| **Covariates** | **AdvSM subgroup** | **HR (95% CI)** | **P value** | **HR (95% CI)** | **P value** |
| **Allo-HCT** | All AdvSM | 1.31 (0.88-1.95) | 0.185 | 1.21 (0.80-1.84) | 0.369 |
|  | SM-AML | 0.19 (0.07-0.54) | **0.002** | 0.20 (0.05-0.78) | **0.020** |
|  | ASM +/- AHN | 1.09 (0.55-2.17) | 0.805 | 1.04 (0.50-2.17) | 0.911 |
|  | MCL +/- AHN | 1.51 (0.67-3.39) | 0.316 | 1.20 (0.48-2.99) | 0.700 |
|  | Other SM-AHN | 4.51 (1.79-11.40) | **0.001** | 2.02 (0.71-5.81) | 0.190 |
| **TKI response** | All AdvSM | 0.64 (0.46-0.91) | **0.013** | 0.42 (0.29-0.61) | **<0.001** |
|  | SM-AML | 1.05 (0.31-3.57) | 0.941 | 2.38 (0.54-10.57) | 0.255 |
|  | ASM +/- AHN | 0.53 (0.34-0.80) | **0.003** | 0.29 (0.18-0.46) | **<0.001** |
|  | MCL +/- AHN | 0.24 (0.07-0.76) | **0.016** | 0.27 (0.08-0.91) | **0.035** |
|  | Other SM-AHN | 1.76 (0.66-4.70) | 0.259 | 0.85 (0.28-2.60) | 0.776 |
| ***KIT* D816V positive** | All AdvSM | 0.72 (0.51-1.00) | 0.052 | 0.68 (0.48-0.96) | **0.027** |
|  | SM-AML | 1.11 (0.33-3.81) | 0.864 | 5.87 (0.88-39.15) | 0.067 |
|  | ASM +/- AHN | 0.94 (0.49-1.78) | 0.843 | 0.84 (0.43-1.65) | 0.608 |
|  | MCL +/- AHN | 0.29 (0.16-0.54) | **<0.001** | 0.46 (0.24-0.88) | **0.018** |
|  | Other SM-AHN | 1.36 (0.52-3.55) | 0.526 | 1.15 (0.41-3.24) | 0.790 |
| **WHO Performance Status** | All AdvSM | 1.76 (1.50-2.07) | **<0.001** | 1.53 (1.27-1.85) | **<0.001** |
|  | SM-AML | 1.09 (0.53-2.25) | 0.815 | 0.72 (0.30-1.77) | 0.475 |
|  | ASM +/- AHN | 1.53 (1.23-1.91) | **<0.001** | 1.54 (1.18-2.00) | **0.002** |
|  | MCL +/- AHN | 1.70 (1.16-2.50) | **0.007** | 1.24 (0.77-1.98) | 0.374 |
|  | Other SM-AHN | 2.19 (1.38-3.47) | **<0.001** | 1.64 (0.98-2.74) | 0.059 |
| **Male sex** | All AdvSM | 1.65 (1.25-2.17) | **<0.001** | 1.34 (1.01-1.78) | **0.043** |
|  | SM-AML | 1.37 (0.58-3.21) | 0.469 | 0.62 (0.18-2.14) | 0.446 |
|  | ASM +/- AHN | 1.72 (1.19-2.47) | **0.004** | 1.77 (1.20-2.61) | **0.004** |
|  | MCL +/- AHN | 1.03 (0.56-1.89) | 0.915 | 0.71 (0.37-1.35) | 0.294 |
|  | Other SM-AHN | 2.98 (1.28-6.93) | **0.011** | 1.97 (0.80-4.88) | 0.140 |
| **IPSM risk group** | All AdvSM | 2.39 (2.04-2.79) | **<0.001** | 2.30 (1.94-2.74) | **<0.001** |
|  | SM-AML | 1.65 (0.94-2.89) | 0.082 | 1.57 (0.81-3.03) | 0.180 |
|  | ASM +/- AHN | 2.34 (1.88-2.91) | **<0.001** | 2.42 (1.93-3.05) | **<0.001** |
|  | MCL +/- AHN | 2.10 (1.48-2.97) | **<0.001** | 2.00 (1.31-3.05) | **0.001** |
|  | Other SM-AHN | 2.79 (1.82-4.28) | **<0.001** | 2.07 (1.33-3.23) | **0.001** |
| **N AdvSM/center** | All AdvSM | 1.08 (1.03-1.13) | **0.003** | 0.96 (0.91-1.01) | 0.123 |
|  | SM-AML | 1.10 (0.86-1.40) | 0.465 | 1.10 (0.82-1.47) | 0.523 |
|  | ASM +/- AHN | 1.03 (0.97-1.10) | 0.321 | 0.91 (0.85-0.98) | **0.012** |
|  | MCL +/- AHN | 1.10 (1.00-1.22) | 0.060 | 0.98 (0.87-1.10) | 0.675 |
|  | Other SM-AHN | 0.88 (0.70-1.10) | 0.256 | 0.91 (0.74-1.11) | 0.346 |
| **Year of diagnosis** | All AdvSM | 0.99 (0.76-1.28) | 0.914 | 1.17 (0.89-1.54) | 0.264 |
|  | SM-AML | 0.45 (0.25-0.80) | **0.007** | 0.34 (0.13-0.92) | **0.034** |
|  | ASM +/- AHN | 1.09 (0.75-1.58) | 0.643 | 1.55 (1.03-2.33) | **0.034** |
|  | MCL +/- AHN | 1.01 (0.54-1.87) | 0.987 | 1.12 (0.53-2.39) | 0.760 |
|  | Other SM-AHN | 2.28 (0.93-5.63) | 0.072 | 1.36 (0.56-3.29) | 0.492 |
| Abbreviations: AdvSM, advanced systemic mastocytosis; AHN, associated hematologic neoplasm; allo-HCT, allogeneic hematopoietic cell transplantation; ASM, aggressive systemic mastocytosis; CI, confidence interval; HR, hazard ratio; IPSM, International Prognostic Scoring System for Mastocytosis; MCL, mast cell leukemia; N, number; SM, systemic mastocytosis; SM-AHN, systemic mastocytosis with an associated hematologic neoplasm; SM-AML, systemic mastocytosis with an associated acute myeloid leukemia; TKI, tyrosine kinase inhibitor; WHO, World Health Organization. | | | | | |

## Supplementary Table 3: Univariate and multivariate Cox proportional hazards regression analysis of prognostic factors for OS in the complete-case AdvSM cohort and for the subtypes SM-AML, ASM +/- AHN, MCL +/- AHN, and other SM-AHN.

Univariate and multivariate Cox proportional hazards regression analysis of prognostic factors for overall survival in the complete-case AdvSM cohort (All AdvSM, *n* = 419, *n* death = 174) and for the diagnostic subgroups SM-AML (*n* = 30, *n* death = 14), ASM +/- AHN (*n* = 223, *n* death = 105), MCL +/- AHN (*n* = 59, *n* death = 34), and other SM-AHN (*n* = 107, *n* death = 21). Allo-HCT treatment (coded as 1 from time of transplantation, 0 otherwise) and TKI response status (coded as 1 from initiation date of first TKI therapy achieving a response, 0 otherwise) were modeled as time-dependent covariates. All other covariates were time-fixed and assessed at time of AdvSM diagnosis. Univariate models assessed each covariate individually. The multivariate model included all pre-specified covariates simultaneously (visualized as a forest plot in **figure 1**). Hazard ratios (HR) >1 indicate increased risk of death; HR <1 indicate reduced risk. For variables modeled per unit increase (WHO performance status per 1-point increase, IPSM risk group per 1-category increase, center experience per 10-patient increase, year of diagnosis per decade), the HR represents the change in hazard per specified increment. *P*-values <0.05 were considered statistically significant.

## **Supplementary Table 4: Univariate and multivariate Cox proportional hazards regression analysis of prognostic factors for OS and PFS in the allo-HCT cohort.**

The multivariate model (1) included only three pre-specified covariates (also visualized as a forest plot in figure 3), whereas multivariate model (2) included the full set of displayed covariates and must be seen as a sensitivity analysis with a high risk of unstable coefficients given the low number of events.

|  | | **Univariate** | | | | **Multivariate Model (1)** | | | | **Multivariate Model (2)** | | | |
| --- | --- | --- | --- | --- | --- | --- | --- | --- | --- | --- | --- | --- | --- |
| **Outcome** | **Variable** | | **N/events** | **HR (95% CI)** | **P value** | | **N/events** | **HR (95% CI)** | **P value** | | **N/events** | **HR (95% CI)** | **P value** |
| **OS** | KPS ≥ 90 | | 57/26 | 0.41 (0.19-0.89) | **0.025** | | 52/24 | 0.37 (0.16-0.85) | **0.020** | | 51/24 | 0.19 (0.06-0.62) | **0.006** |
|  | IPSM risk group at allo-HCT | | 65/33 | 1.91 (1.29-2.84) | **0.001** | | 52/24 | 1.68 (1.07-2.65) | **0.026** | | 51/24 | 1.46 (0.83-2.57) | 0.184 |
|  | CR/PR before allo-HCT | | 65/30 | 0.41 (0.19-0.88) | **0.022** | | 52/24 | 0.55 (0.23-1.30) | 0.175 | | 51/24 | 0.73 (0.25-2.11) | 0.558 |
|  | Sex | | 69/33 | 2.22 (0.96-5.11) | 0.062 | |  |  |  | | 51/24 | 3.51 (0.89-13.94) | 0.074 |
|  | MAC conditioning | | 66/31 | 0.79 (0.38-1.65) | 0.528 | |  |  |  | | 51/24 | 1.47 (0.49-4.40) | 0.490 |
|  | *KIT* D816V mutation | | 68/33 | 0.55 (0.25-1.23) | 0.148 | |  |  |  | | 51/24 | 1.76 (0.57-5.39) | 0.325 |
|  | Any prior TKI treatment response | | 69/33 | 1.29 (0.65-2.56) | 0.462 | |  |  |  | | 51/24 | 1.64 (0.61-4.44) | 0.330 |
|  | AdvSM subtype (vs. Other SM-AHN) | |  |  |  | |  |  |  | |  |  |  |
|  | ASM +/- AHN | | 69/33 | 0.95 (0.37-2.47) | 0.923 | |  |  |  | | 51/24 | 0.65 (0.16-2.67) | 0.555 |
|  | MCL +/- AHN | | 69/33 | 1.22 (0.44-3.37) | 0.700 | |  |  |  | | 51/24 | 2.24 (0.61-8.23) | 0.226 |
|  | SM-AML | | 69/33 | 0.68 (0.24-1.95) | 0.474 | |  |  |  | | 51/24 | 0.48 (0.13-1.76) | 0.272 |
| **PFS** | KPS ≥ 90 | | 55/30 | 0.57 (0.28-1.18) | 0.130 | | 50/28 | 0.56 (0.26-1.21) | 0.140 | | 49/28 | 0.37 (0.13-1.02) | 0.055 |
|  | IPSM risk group at allo-HCT | | 63/38 | 1.92 (1.32-2.79) | **<0.001** | | 50/28 | 1.99 (1.27-3.11) | **0.003** | | 49/28 | 1.79 (1.03-3.10) | **0.037** |
|  | CR/PR before allo-HCT | | 63/35 | 0.40 (0.20-0.81) | **0.011** | | 50/28 | 0.55 (0.25-1.23) | 0.148 | | 49/28 | 0.70 (0.26-1.92) | 0.493 |
|  | Sex | | 67/38 | 2.04 (0.97-4.32) | 0.061 | |  |  |  | | 49/28 | 4.03 (1.03-15.74) | **0.045** |
|  | MAC conditioning | | 64/36 | 0.56 (0.29-1.10) | 0.092 | |  |  |  | | 49/28 | 1.01 (0.37-2.76) | 0.981 |
|  | *KIT* D816V mutation | | 66/37 | 0.62 (0.28-1.37) | 0.239 | |  |  |  | | 49/28 | 2.54 (0.80-8.04) | 0.113 |
|  | Any prior TKI treatment response | | 67/38 | 0.89 (0.47-1.70) | 0.734 | |  |  |  | | 49/28 | 1.04 (0.41-2.61) | 0.940 |
|  | AdvSM subtype (vs. Other SM-AHN) | |  |  |  | |  |  |  | |  |  |  |
|  | ASM +/- AHN | | 67/38 | 1.03 (0.41-2.64) | 0.943 | |  |  |  | | 49/28 | 0.86 (0.23-3.29) | 0.827 |
|  | MCL +/- AHN | | 67/38 | 1.83 (0.68-4.93) | 0.230 | |  |  |  | | 49/28 | 4.29 (1.10-16.72) | **0.036** |
|  | SM-AML | | 67/38 | 1.08 (0.41-2.83) | 0.883 | |  |  |  | | 49/28 | 0.61 (0.18-2.01) | 0.413 |
| Abbreviations: AdvSM, advanced systemic mastocytosis; allo-HCT, allogeneic hematopoietic cell transplantation; ASM, aggressive systemic mastocytosis; CI, confidence interval; HR, hazard ratio; CR, complete remission; IPSM, International Prognostic Scoring System for Mastocytosis; KPS, Karnofsky Performance Status; MAC, myeloablative conditioning; MCL, mast cell leukemia; OS, overall survial; PFS, progression-free survival; PR, partial remission; SM, systemic mastocytosis; SM-AHN, systemic mastocytosis with an associated hematologic neoplasm; SM-AML, systemic mastocytosis with an associated acute myeloid leukemia; TKI, tyrosine kinase inhibitor; WHO, World Health Organization. | | | | | | | | | | | | | |

## Supplementary Table 5: Univariate and multivariate Cox proportional hazards regression analysis of prognostic factors including the MARS for OS and PFS in the allo-HCT cohort.

|  | | **Univariate** | | | **Multivariate Model** | | | | |
| --- | --- | --- | --- | --- | --- | --- | --- | --- | --- |
| **Endpoint** | **Variable** | **N/events** | **HR (95% CI)** | **P value** | **N/events** | **HR (95% CI)** | **P value** | |  |
| **OS** | KPS ≥ 90 | 57/26 | 0.41 (0.19-0.89) | **0.025** | 43/21 | 0.32 (0.13-0.79) | **0.014** |  |  |
|  | MARS risk group at allo-HCT | 48/23 | 1.54 (0.94-2.52) | 0.090 | 43/21 | 1.50 (0.89-2.53) | 0.128 |  |  |
|  | CR/PR before allo-HCT | 65/30 | 0.41 (0.19-0.88) | **0.022** | 43/21 | 0.37 (0.15-0.94) | **0.037** |  |  |
| **PFS** | KPS ≥ 90 | 55/30 | 0.57 (0.28-1.18) | 0.130 | 41/23 | 0.42 (0.18-1.00) | 0.050 |  |  |
|  | MARS risk group at allo-HCT | 46/25 | 1.53 (0.94-2.50) | 0.086 | 41/23 | 1.64 (0.98-2.73) | 0.058 |  |  |
|  | CR/PR before allo-HCT | 63/35 | 0.40 (0.20-0.81) | **0.011** | 41/23 | 0.46 (0.19-1.07) | 0.072 |  |  |
| Abbreviations: Allo-HCT, allogeneic hematopoietic cell transplantation; CI, confidence interval; CR, complete remission; HR, hazard ratio; KPS, Karnofsky Performance Status; MARS, mutation-adjusted risk score; OS, overall survival; PFS, progression-free survival; PR, partial remission. | | | | | | | |  |  |

|  | **All AdvSM** | **AdvSM complete-cases*** | **P value** |
| --- | --- | --- | --- |
|  | *N =* *631* | *N = 419* |  |
| **Age**, years, median (IQR, N) | 64 (54-71, 631) | 62 (52-68, 419) | **<0.001** |
| **Sex (male)**, %, (N) | 62 (394/631) | 59 (249/419) | **0.030** |
| **WHO Performance Status**, median (IQR, N) | 1 (1-2, 591) | 1 (1-2, 419) | 0.358 |
| **Follow up (months),** median (IQR, N) | 31.2 (12.2-58.5, 631) | 35.7 (15.6-64.5, 419) | **<0.001** |
| **AdvSM subtypes** |  | | |
| - ASM±AHN % (N) | 56 (354/631) | 53 (223/419) | **0.042** |
| - MCL±AHN % (N) | 12 (74/631) | 14 (59/419) | **0.009** |
| - SM-AML % (N) | 6 (41/631) | 7 (30/419) | 0.396 |
| - Other SM-AHN % (N) | 26 (162/631) | 26 (107/419) | 0.923 |
| **IPSM Score,** median (IQR, N) | 3 (2-3, 569) | 3 (2-3, 419) | **<0.001** |
| - Risk group 1 % (N) | 19 (110/569) | 22 (92/419) | **0.008** |
| - Risk group 2 % (N) | 20 (115/569) | 23 (95/419) | **0.017** |
| - Risk group 3 % (N) | 46 (260/569) | 41 (173/419) | **<0.001** |
| - Risk group 4 N % (N) | 15 (84/569) | 14 (59/419) | 0.502 |
| ***KIT* D816V positive,** % (N) | 86 (509/589) | 85 (357/419) | 0.187 |
| **Aberrant Karyotype,** % (N) | 19 (63/337) | 18 (46/252) | 0.748 |
| **S/A/R status positive,** % (N) | 43 (75/173) | 44 (55/126) | 1.000 |
| **Lab values at AdvSM diagnosis** |  | | |
| - Serum Tryptase, µg/L, median (IQR, N) | 148 (56-256, 585) | 147 (56-269, 419) | 0.887 |
| - WBC, G/L, median (IQR, N) | 8 (4.65-13.44, 618) | 7.8 (4.6-12.9, 419) | 0.162 |
| - Eosinophils, G/L, median (IQR, N) | 0.2 (0.05-0.79, 546) | 0.18 (0.04-0.71, 382) | 0.359 |
| - Hemoglobin, g/dL, median (IQR, N) | 10.9 (9.3-12.8, 615) | 11 (9.3-12.9, 419) | 0.254 |
| - Platelets, G/L, median (IQR, N) | 140 (77.5-254.5, 615) | 158 (80-264, 419) | **0.045** |
| - LDH, U/L, median (IQR, N) | 177 (140-253, 537) | 172 (136-234, 381) | **0.004** |
| - AP, U/L, median (IQR, N) | 150.5 (88-290, 556) | 136.5 (83.87-275, 386) | 0.072 |
| **Cytoreductive treatment** |  | | |
| - TKI, % (N) | 51 (320/631) | 54 (227/419) | **0.012** |
| - Allo-HCT, % (N) | 11 (69/631) | 16 (66/419) | **<0.001** |
| - Polychemotherapy, % (N) | 13 (83/631) | 15 (62/419) | 0.105 |
| - 2-CdA ,% (N) | 26 (164/631) | 27 (115/419) | 0.251 |
| - IFN-α, % (N) | 14 (91/631) | 16 (65/419) | 0.284 |
| - Hydroxyurea, % (N) | 16 (103/631) | 17 (70/419) | 0.820 |
| - NA, % (N) | 22 (138/631) | 18 (77/419) | **0.004** |
| Abbreviations: 2-CdA, Cladribine; AdvSM, advanced systemic mastocytosis; AHN, associated hematologic neoplasm; allo-HCT, allogeneic hematopoietic cell transplantation; AP, alkaline phosphatase; ASM, aggressive systemic mastocytosis; IFN-α, interferon alpha; IQR, interquartile range; IPSM, international prognostic scoring system in mastocytosis; LDH, lactate dehydrogenase; m, male; MCL, mast cell leukemia; n, number; NA, not available; S/A/R, *SRSF2*/*ASXL1*/*RUNX1*; SM-AHN, systemic mastocytosis with an associated hematologic neoplasm; SM-AML, systemic mastocytosis with an associated acute myeloid leukemia; TKI, tyrosine kinase inhibitor (includes midostaurin, avapritinib, imatinib, and investigational TKIs); WBC, white blood cells; WHO, world health organization.  *This comparison contrasts the primary AdvSM cohort with the complete-case cohort used for the OS and PFS analyses. Observations here were not censored at 60 months after SM diagnosis, in order to provide an unbiased comparison of the two cohorts. Accordingly, 66 patients in the complete-case cohort are shown as having undergone allo-HCT, whereas Supplementary Table 7 lists 63, because three patients were transplanted more than 60 months after diagnosis and were therefore censored before transplantation in the respective analyses. | | | |

## Supplementary Table 6. Patient characteristics of the primary and complete-case AdvSM cohort.

## **Supplementary Table 7: Comparison of baseline characteristics between patients who received allo-HCT and those who did not (complete-case cohort).**

|  | **No Allo-HCT** | **Allo-HCT** | **P value** |
| --- | --- | --- | --- |
|  | *N* = 356 | *N* = 63 |  |
| **Sex (male)**, %, (N) | 58 (205/356) | 70 (44/63) | 7.18E-02 |
| **Age**, years, median (IQR, N) | 63.5 (54-69, 356) | 54 (47-60, 63) | **2.72E-08** |
| **WHO Performance Status**, median (IQR, N) | 1 (1-2, 356) | 1 (1-2, 63) | 7.77E-01 |
| **Follow up (months),** median (IQR, N) | 34.9 (13.81-60, 356) | 41.6 (24.72-60, 63) | 8.21E-02 |
| **AdvSM subtypes** |  | | |
| - SM-AML % (N) | 3 (12/356) | 29 (18/63) | **3.35E-09** |
| - MCL±AHN % (N) | 13 (47/356) | 19 (12/63) | 2.38E-01 |
| - ASM±AHN % (N) | 57 (202/356) | 33 (21/63) | **9.00E-04** |
| - Other SM-AHN % (N) | 27 (95/356) | 19 (12/63) | 2.15E-01 |
| **IPSM Score,** median (IQR, N) | 3 (2-3, 356) | 3 (2-3, 63) | 4.93E-01 |
| - Risk group 1 % (N) | 22 (77/356) | 24 (15/63) | 7.41E-01 |
| - Risk group 2 % (N) | 22 (80/356) | 24 (15/63) | 8.70E-01 |
| - Risk group 3 % (N) | 41 (147/356) | 41 (26/63) | 1.00E+00 |
| - Risk group 4 N % (N) | 15 (52/356) | 11 (7/63) | 5.59E-01 |
| ***KIT* D816V positive,** % (N) | 85 (304/356) | 84 (53/63) | 8.47E-01 |
| **Aberrant Karyotype,** % (N) | 12 (24/196) | 39 (22/56) | **1.79E-05** |
| **S/A/R status positive,** % (N) | 42 (33/79) | 47 (22/47) | 7.11E-01 |
| **Lab values at AdvSM diagnosis** |  | | |
| - Serum Tryptase, µg/L, median (IQR, N) | 153.5 (56.22-274.25, 356) | 97.2 (43-200, 63) | **4.15E-02** |
| - WBC, G/L, median (IQR, N) | 8 (4.89-12.93, 356) | 6.6 (3.15-12.1, 63) | 8.49E-02 |
| - Eosinophils, G/L, median (IQR, N) | 0.19 (0.05-0.69, 329) | 0.15 (0.02-0.88, 53) | 4.58E-01 |
| - Hemoglobin, g/dL, median (IQR, N) | 11.1 (9.6-13, 356) | 9.7 (8.45-12.1, 63) | **2.24E-03** |
| - Platelets, G/L, median (IQR, N) | 172.5 (83-283.75, 356) | 111 (61.5-219, 63) | **1.72E-03** |
| - LDH, U/L, median (IQR, N) | 167.8 (131.25-227, 322) | 190 (160-334.5, 59) | **7.32E-04** |
| - AP, U/L, median (IQR, N) | 137 (83.62-300.3, 324) | 134.5 (90.11-211.55, 62) | 4.08E-01 |
| **Cytoreductive treatment** |  | | |
| - TKI, % (N) | 54 (192/356) | 57 (36/63) | 6.82E-01 |
| - Allo-HCT, % (N) | 0 (0/356) | 100 (63/63) | **1.70E-76** |
| - Polychemotherapy, % (N) | 11 (38/356) | 38 (24/63) | **4.11E-07** |
| - 2-CdA, % (N) | 27 (96/356) | 30 (19/63) | 6.46E-01 |
| - IFN-α, % (N) | 16 (58/356) | 11 (7/63) | 3.49E-01 |
| - Hydroxyurea, % (N) | 17 (62/356) | 13 (8/63) | 4.64E-01 |
| - NA, % (N) | 22 (77/356) | 0 (0/63) | **1.48E-06** |
| Continuous variables compared using Wilcoxon rank-sum test; categorical variables compared using Fisher's exact test.  Abbreviations: 2-CdA, Cladribine; AdvSM, advanced systemic mastocytosis; AHN, associated hematologic neoplasm; allo-HCT, allogeneic hematopoietic cell transplantation; AP, alkaline phosphatase; ASM, aggressive systemic mastocytosis; IFN-α, interferon alpha; IQR, interquartile range; IPSM, international prognostic scoring system in mastocytosis; LDH, lactate dehydrogenase; m, male; MCL, mast cell leukemia; n, number; NA, not available; S/A/R, *SRSF2*/*ASXL1*/*RUNX1*; SM-AHN, systemic mastocytosis with an associated hematologic neoplasm; SM-AML, systemic mastocytosis with an associated acute myeloid leukemia; TKI, tyrosine kinase inhibitor; WBC, white blood cells; WHO, world health organization. | | | |

## Supplementary Table 8. Patient characteristics of the allo-HCT cohort.

|  | **All subtypes** | **ASM±AHN** | **MCL±AHN** | **SM-AML** | **Other SM-AHN** |
| --- | --- | --- | --- | --- | --- |
|  | *N = 69* | *N = 23* | *N = 13* | *N = 19* | *N = 14* |
| **Age**, years, median (IQR, N) | 53 (46-60, 69) | 54 (47-59, 23) | 45 (36-50, 13) | 59 (55-62.5, 19) | 51.5 (46.25-57.25, 14) |
| **Age at allo-HCT**, years, median (IQR, N) | 55 (47-61, 69) | 58 (48-62, 23) | 45 (37-52, 13) | 61 (55.5-63, 19) | 53.5 (47-60.25, 14) |
| **Sex (male)**, %, (N) | 67 (46) | 74 (17/23) | 54 (7/13) | 63 (12/19) | 71 (10/14) |
| **Karnofsky performance status at time of allo-HCT**, median (IQR, N) | 90 (80-90, 57) | 85 (80-90, 17) | 90 (80-90, 10) | 90 (85-100, 17) | 90 (80-90, 13) |
| **Follow up after allo-HCT (months),**  median (IQR, N) | 26.2 (8.3-51.7, 69) | 28.2 (3.8-58.7, 23) | 13 (9.7-92.4, 13) | 32.4 (17.7-46.5, 19) | 17.8 (7.7-44.2, 14) |
| **IPSM Score at allo-HCT,** median (IQR, N) | 2.5 (1.25-3, 66) | 2 (1.5-3, 23) | 2 (2-3, 12) | 3 (2-3, 18) | 2 (1-3, 13) |
| - Risk group 1 % (N) | 26 (17/66) | 26 (6/23) | 17 (2/12) | 17 (3/18) | 46 (6/13) |
| - Risk group 2 % (N) | 24 (16/66) | 26 (6/23) | 42 (5/12) | 17 (3/18) | 15 (2/13) |
| - Risk group 3 % (N) | 39 (26/66) | 39 (9/23) | 33 (4/12) | 50 (9/18) | 31 (4/13) |
| - Risk group 4 % (N) | 11 (7/66) | 9 (2/23) | 8 (1/12) | 17 (3/18) | 8 (1/13) |
| **Prior therapies** |  |  |  |  |  |
| - N lines of therapy prior to allo-HCT, median (IQR, N) | 2 (1-3, 55) | 2 (1-3, 19) | 2.5 (1-3, 12) | 1.5 (1-2, 16) | 1.5 (1-2, 8) |
| - Midostaurin, % (N) | 43 (30/69) | 52 (12/23) | 62 (8/13) | 37 (7/19) | 21 (3/14) |
| - Polychemotherapy, % (N) | 35 (24/69) | 9 (2/23) | 46 (6/13) | 47 (9/19) | 50 (7/14) |
| - 2-CdA, % (N) | 32 (22/69) | 48 (11/23) | 54 (7/13) | 11 (2/19) | 14 (2/14) |
| - IFN-α, % (N) | 13 (9/69) | 26 (6/23) | 23 (3/13) | 0 (0/19) | 0 (0/14) |
| - Ruxolitinib, % (N) | 6 (4/69) | 9 (2/23) | 8 (1/13) | 0 (0/19) | 7 (1/14) |
| - Imatinib/Dasatinib/Nilotinib, % (N) | 7 (5/69) | 9 (2/23) | 15 (2/13) | 5 (1/19) | 0 (0/14) |
| - Hydroxyurea, % (N) | 6 (4/69) | 9 (2/23) | 0 (0/13) | 5 (1/19) | 7 (1/14) |
| - Hypomethylating Agents, % (N) | 9 (6/69) | 9 (2/23) | 0 (0/13) | 11 (2/19) | 14 (2/14) |
| - Other cytoreductive therapy, % (N) | 6 (4/69) | 0 (0/23) | 15 (2/13) | 0 (0/19) | 14 (2/14) |
| - None (upfront allo-HCT), % (N) | 4 (3/69) | 4 (1/23) | 8 (1/13) | 5 (1/19) | 0 (0/14) |
| - NA, % (N) | 10 (7/69) | 13 (3/23) | 0 (0/13) | 5 (1/19) | 21 (3/14) |
| **Remission at allo-HCT** |  |  |  |  |  |
| - CR/PR before Allo-HCT, % (N) | 46 (30/65) | 50 (10/20) | 31 (4/13) | 53 (10/19) | 46 (6/13) |
| **Stem cell source** |  |  |  |  |  |
| - Peripheral blood, % (N) | 95 (61/64) | 100 (20/20) | 83 (10/12) | 94 (17/18) | 100 (14/14) |
| - Bone marrow, % (N) | 5 (3/64) | 0 (0/20) | 17 (2/12) | 6 (1/18) | 0 (0/14) |
| **Type of donor** |  |  |  |  |  |
| - Related donor, % (N) | 44 (30/68) | 36 (8/22) | 54 (7/13) | 42 (8/19) | 50 (7/14) |
| - Unrelated donor, % (N) | 56 (38/68) | 64 (14/22) | 46 (6/13) | 58 (11/19) | 50 (7/14) |
| **HLA matching** |  |  |  |  |  |
| - Matched, % (N) | 74 (48/65) | 75 (15/20) | 77 (10/13) | 78 (14/18) | 64 (9/14) |
| - Haploidentical, % (N) | 9 (6/65) | 5 (1/20) | 15 (2/13) | 6 (1/18) | 14 (2/14) |
| - Partially matched, % (N) | 17 (11/65) | 20 (4/20) | 8 (1/13) | 17 (3/18) | 21 (3/14) |
| **Conditioning** |  |  |  |  |  |
| - Myeloablative, % (N) | 70 (46/66) | 71 (15/21) | 54 (7/13) | 67 (12/18) | 86 (12/14) |
| - Reduced intensity, % (N) | 30 (20/66) | 29 (6/21) | 46 (6/13) | 33 (6/18) | 14 (2/14) |
| **GVHD Prophylaxis** |  |  |  |  |  |
| - CsA, % (N) | 67 (46/69) | 52 (12/23) | 62 (8/13) | 84 (16/19) | 71 (10/14) |
| - ATG, % (N) | 35 (24/69) | 30 (7/23) | 46 (6/13) | 42 (8/19) | 21 (3/14) |
| - MTX, % (N) | 33 (23/69) | 35 (8/23) | 31 (4/13) | 26 (5/19) | 43 (6/14) |
| - MMF, % (N) | 36 (25/69) | 26 (6/23) | 38 (5/13) | 42 (8/19) | 43 (6/14) |
| - Tacrolimus, % (N) | 14 (10/69) | 13 (3/23) | 15 (2/13) | 16 (3/19) | 14 (2/14) |
| - Post-Tx Cyclophosphamide, % (N) | 12 (8/69) | 13 (3/23) | 8 (1/13) | 16 (3/19) | 7 (1/14) |
| - Alemtuzumab (Campath®), % (N) | 6 (4/69) | 4 (1/23) | 8 (1/13) | 5 (1/19) | 7 (1/14) |
| - NA, % (N) | 13 (9/69) | 22 (5/23) | 15 (2/13) | 5 (1/19) | 7 (1/14) |
| **Engraftment (days post allo-HCT)** |  |  |  |  |  |
| - Neutrophils, ≥0.5 G/L, median (IQR, N) | 18 (15-22, 57) | 16 (14.75-19, 16) | 18 (14.5-21, 11) | 19 (17-22.25, 18) | 19.5 (14.5-22, 12) |
| - Platelets, ≥20 G/L, median (IQR, N) | 19 (13-25.5, 50) | 16.5 (13.25-24.5, 14) | 23 (19.5-37, 10) | 19 (12-22, 15) | 19 (15-23, 11) |
| **Acute GVHD**, % (N) | 51 (35/69) | 52 (12/23) | 54 (7/13) | 53 (10/19) | 43 (6/14) |
| **Chronic GVHD**, % (N) | 25 (17/69) | 30 (7/23) | 15 (2/13) | 21 (4/19) | 29 (4/14) |
| **Outcome** |  |  |  |  |  |
| - Relapse, % (N) | 61 (42/69) | 52 (12/23) | 77 (10/13) | 63 (12/19) | 57 (8/14) |
| - Death, % (N) | 52 (36/69) | 48 (11/23) | 62 (8/13) | 47 (9/19) | 57 (8/14) |
| - RRM, % (N) | 44 (16/36) | 27 (3/11) | 62 (5/8) | 33 (3/9) | 62 (5/8) |
| - TRM, % (N) | 39 (14/36) | 55 (6/11) | 25 (2/8) | 56 (5/9) | 12 (1/8) |
| - Other cause of death, % (N) | 17 (6/36) | 18 (2/11) | 12 (1/8) | 11 (1/9) | 25 (2/8) |
| Abbreviations: 2-CdA, Cladribine; AdvSM, advanced systemic mastocytosis; AHN, associated hematologic neoplasm; allo-HCT, allogeneic hematopoietic cell transplantation; ASM, aggressive systemic mastocytosis; ATG, Anti-thymocyte globulin; CsA, cyclosporine A; GVHD, graft-versus-host disease; HLA, human leukocyte antigen; HU, hydroxyurea; IFN-α, interferon alpha; IQR, interquartile range; IPSM, international prognostic scoring system in mastocytosis; m, male; MCL, mast cell leukemia; MMF, m**ycophenolate mofetil**; MTX, methotrexate; N, number; NA, not available; RRM, relapse-related mortality; S/A/R, *SRSF2*/*ASXL1*/*RUNX1*; SM-AHN, systemic mastocytosis with an associated hematologic neoplasm; SM-AML, systemic mastocytosis with an associated acute myeloid leukemia; TRM, transplant-related mortality. | | | | | |

## Supplementary Table 9: Cumulative incidence of competing causes of death after allo-HCT.

| **Event Type** | **N/events** | **Year 2** | **Year 5** |
| --- | --- | --- | --- |
| RRM | 69/16 | 18% (9.6%, 28%) | 26% (15%, 37%) |
| TRM | 69/12 | 16% (8.6%, 26%) | 18% (9.9%, 29%) |
| Other cause of death | 69/5 | 2.9% (0.54%, 9.1%) | 10% (3.5%, 21%) |
| Abbreviations: Allo-HCT, allogeneic hematopoietic stem cell transplantation; N, number; RRM, relapse-related mortality; TRM, transplant-related mortality. | | | |

## Supplementary Table 10: Cumulative incidence of competing causes of death after allo-HCT: detailed estimates stratified by combined IPSM risk groups.

| **Event Type** | **N/events** | **Year 2** | **Year 5** | **P value** |
| --- | --- | --- | --- | --- |
| **RRM** |  |  |  | 0.5 |
| 1 + 2 | 34/7 | 18% (7.1%, 33%) | 22% (9.3%, 38%) |  |
| 3 + 4 | 31/9 | 19% (7.6%, 35%) | 33% (15%, 51%) |  |
| **TRM** |  |  |  | 0.12 |
| 1 + 2 | 34/4 | 9.7% (2.3%, 23%) | 14% (4.1%, 29%) |  |
| 3 + 4 | 31/8 | 26% (12%, 42%) | 26% (12%, 42%) |  |
| **Other cause of death** |  |  |  | 0.018 |
| 1 + 2 | 34/0 | 0.00% (—%, —%) | 0.00% (—%, —%) |  |
| 3 + 4 | 31/5 | 6.5% (1.1%, 19%) | 22% (7.1%, 41%) |  |
| P-value from Gray's test  Abbreviations: Allo-HCT, allogeneic hematopoietic stem cell transplantation; IPSM, International Prognostic Scoring System for Mastocytosis; N, number; RRM, relapse-related mortality; TRM, transplant-related mortality. | | | | |

## Supplementary Table 11: Univariate and multivariate Cox proportional hazards regression analysis of the individual variables contained in the IPSM scoring system on OS and PFS in the allo-HCT cohort.

|  | | **Univariate** | | | **Multivariate Model** | | |
| --- | --- | --- | --- | --- | --- | --- | --- |
| **Endpoint** | **Variable** | **N/events** | **HR (95% CI)** | **P value** | **N/events** | **HR (95% CI)** | **P value** |
| **OS** | Age ≥ 60 | 69/33 | 2.26 (1.13-4.52) | **0.021** | 58/29 | 2.21 (1.01-4.86) | **0.047** |
|  | Tryptase ≥ 125ng/mL | 61/29 | 2.75 (1.32-5.74) | **0.007** | 58/29 | 3.33 (1.52-7.29) | **0.003** |
|  | Hb ≤ 11g/dL | 65/33 | 1.39 (0.68-2.83) | 0.367 | 58/29 | 0.58 (0.24-1.39) | 0.220 |
|  | WBC ≥ 16G/L | 65/33 | 3.74 (1.71-8.18) | **<0.001** | 58/29 | 4.64 (1.93-11.11) | **<0.001** |
|  | Skin involvement | 68/33 | 1.32 (0.64-2.73) | 0.453 | 58/29 | 1.17 (0.53-2.62) | 0.697 |
|  | Platelets ≤ 100G/L | 65/33 | 1.76 (0.87-3.54) | 0.114 | 58/29 | 1.34 (0.59-3.03) | 0.488 |
| **PFS** | Age ≥ 60 | 67/38 | 2.47 (1.29-4.73) | **0.006** | 57/32 | 2.87 (1.36-6.07) | **0.006** |
|  | Tryptase ≥ 125ng/mL | 60/32 | 2.46 (1.22-4.96) | **0.012** | 57/32 | 2.67 (1.27-5.62) | **0.009** |
|  | Hb ≤ 11g/dL | 63/38 | 1.30 (0.67-2.52) | 0.435 | 57/32 | 0.64 (0.28-1.45) | 0.283 |
|  | WBC ≥ 16G/L | 63/38 | 2.77 (1.29-5.95) | **0.009** | 57/32 | 3.32 (1.44-7.68) | **0.005** |
|  | Skin involvement | 66/38 | 1.33 (0.67-2.63) | 0.419 | 57/32 | 1.05 (0.49-2.23) | 0.909 |
|  | Platelets ≤ 100G/L | 63/38 | 1.84 (0.96-3.55) | 0.067 | 57/32 | 1.61 (0.72-3.59) | 0.248 |
| Abbreviations: Allo-HCT, allogeneic hematopoietic cell transplantation; CI, confidence interval; CR, complete remission; Hb, hemoglobin; HR, hazard ratio; IPSM, International Prognostic Scoring System for Mastocytosis; KPS, Karnofsky Performance Status; OS, overall survival; PFS, progression-free survival; PR, partial remission; WBC, white blood cell count. | | | | | | | |

## Supplementary Table 12: TKI treatment characteristics of the AdvSM cohort

| **Substance** | **Treated patients (N)** | **Single therapy** | **Sequential therapy** | **Most frequent sequence of TKI treatments** (ordered by start date of therapy, n = patients) |
| --- | --- | --- | --- | --- |
| Midostaurin | 264 | 221 (83.7%) | 43 (16.3%) | Midostaurin, Avapritinib (*n = 14*) |
| Imatinib | 40 | 23 (57.5%) | 17 (42.5%) | Imatinib, Midostaurin (*n = 9*) |
| Avapritinib | 18 | 2 (11.1%) | 16 (88.9%) | Midostaurin, Avapritinib (*n = 14*) |
| Dasatinib | 14 | 6 (42.9%) | 8 (57.1%) | Dasatinib, Midostaurin (*n = 4*) |
| Ruxolitinib | 12 | 5 (41.7%) | 7 (58.3%) | Ruxolitinib, Midostaurin (*n = 5*) |
| Ibrutinib | 5 | 4 (80.0%) | 1 (20.0%) | Ibrutinib, Midostaurin (*n = 1*) |
| Nilotinib | 4 | 1 (25.0%) | 3 (75.0%) | Dasatinib, Nilotinib, Sorafenib (*n = 1*) |
| Masitinib | 2 | 1 (50.0%) | 1 (50.0%) | Midostaurin, Masitinib (*n = 1*) |
| Ponatinib | 1 | 0 (0.0%) | 1 (100.0%) | Imatinib, Dasatinib, Ponatinib (*n = 1*) |
| Ripretinib | 1 | 0 (0.0%) | 1 (100.0%) | Midostaurin, Ripretinib (*n = 1*) |
| Sorafenib | 1 | 0 (0.0%) | 1 (100.0%) | Dasatinib, Nilotinib, Sorafenib (*n = 1*) |
| Note: Overall, 319 of 631 (51%) in the primary AdvSM cohort received TKI treatment. For this descriptive analysis, we used all patients from the full primary AdvSM cohort, which was not filtered for complete cases, age, or WHO performance status. In addition, no administrative censoring was performed. Any given TKI is shown independent of the indication. | | | | |

## Supplementary Table 13 A: TKI treatment characteristics of the allo-HCT cohort

| **Substance** | **Treated patients (N)** | **Single therapy** | **Sequential therapy** | **Most frequent sequence of TKI treatments** (ordered by start date of therapy, n = patients) |
| --- | --- | --- | --- | --- |
| Midostaurin | 33 | 26 (78.8%) | 7 (21.2%) | Ruxolitinib, Midostaurin (*n = 2*) |
| Ruxolitinib | 5 | 3 (60.0%) | 2 (40.0%) | Ruxolitinib, Midostaurin (*n = 2*) |
| Imatinib | 4 | 1 (25.0%) | 3 (75.0%) | Imatinib, Midostaurin (*n = 1*) |
| Dasatinib | 2 | 1 (50.0%) | 1 (50.0%) | Dasatinib, Nilotinib, Sorafenib (*n = 1*) |
| Nilotinib | 2 | 0 (0.0%) | 2 (100.0%) | Dasatinib, Nilotinib, Sorafenib (*n = 1*) |
| Avapritinib | 1 | 0 (0.0%) | 1 (100.0%) | Imatinib, Midostaurin, Avapritinib (*n = 1*) |
| Masitinib | 1 | 0 (0.0%) | 1 (100.0%) | Masitinib, Midostaurin (*n = 1*) |
| Sorafenib | 1 | 0 (0.0%) | 1 (100.0%) | Dasatinib, Nilotinib, Sorafenib (*n = 1*) |
| Note: Overall, 37 of 69 (54%) allo-HCT-treated patients in the primary AdvSM cohort received TKI treatment. For this descriptive analysis, we used all allo-HCT-treated patients from the full primary AdvSM cohort, which was not filtered for complete cases, age, or WHO performance status. In addition, no administrative censoring was performed. Any given TKI is shown independent of the indication. | | | | |

## Supplementary Table 13 B: TKI treatment before and after allo-HCT

| **Substance** | **Given before allo-HCT**  (N patients) | **Given before allo-HCT**  (N patients) | **Total**  (N patients) |
| --- | --- | --- | --- |
| Any TKI | 31 | 7 | 37 |
| Midostaurin | 29 | 3 | 32 |
| Ruxolitinib | 4 | 1 | 5 |
| Imatinib | 3 | 1 | 4 |
| Nilotinib | 1 | 1 | 2 |
| Avapritinib | 0 | 1 | 1 |
| Dasatinib | 0 | 1 | 1 |
| Sorafenib | 0 | 1 | 1 |
| Note: Overall, 37 of 69 (54%) allo-HCT-treated patients in the primary AdvSM cohort received TKI treatment. For this descriptive analysis, we used all allo-HCT-treated patients from the full primary AdvSM cohort, which was not filtered for complete cases, age, or WHO performance status. In addition, no administrative censoring was performed. Any given TKI is shown independent of the indication. | | | |

## Supplementary Table 14: Characteristics of SM-AML patients included in comparative analyses for allo-HCT and TKI-treatment efficacy (*n = 30*).

|  | **Allo-HCT** | **No allo-HCT** |
| --- | --- | --- |
|  | *N =* *18* | *N = 12* |
| **Sex (male),** % (N) | 61 (11/18) | 42 (5/12) |
| **Age (years),** median (IQR, N) | 59 (54.5-62.75, 18) | 64.5 (58-67.25, 12) |
| **WHO Performance Status,** median (IQR, N) | 1 (1-1, 18) | 1 (1-1.25, 12) |
| **IPSM Score,** median (IQR, N) | 3 (2-3, 18) | 3 (2-3, 12) |
| - group 1, % (N) | 17 (3/18) | 8 (1/12) |
| - group 2, % (N) | 17 (3/18) | 25 (3/12) |
| - group 3, % (N) | 50 (9/18) | 50 (6/12) |
| - group 4, % (N) | 17 (3/18) | 17 (2/12) |
| **KIT-D816V positive,** % (N) | 94 (17/18) | 67 (8/12) |
| **Karyotype aberrant,** % (N) | 59 (10/17) | 45 (5/11) |
| **S/A/R status positive,** % (N) | 64 (9/14) | 0 (0/3) |
| **Lab values at diagnosis** |  |  |
| - Serum Tryptase, µg/L, median (IQR, N) | 77.7 (23.62-229.25, 18) | 66.2 (14.27-152.5, 12) |
| - WBC, G/L, median (IQR, N) | 4.2 (1.93-11.75, 18) | 4 (1.25-8.4, 12) |
| - Eosinophils, G/L, median (IQR, N) | 0.9 (0-26, 15) | 1 (0-22.01, 10) |
| - Hemoglobin, g/dL, median (IQR, N) | 9.6 (7.8-11.28, 18) | 9.2 (6.95-11.57, 12) |
| - Platelets, G/L, median (IQR, N) | 54.5 (30.5-105.75, 18) | 81 (49.5-184.25, 12) |
| - LDH, U/L, median (IQR, N) | 265.5 (208.75-388, 16) | 181.5 (127.75-550, 10) |
| - AP, U/L, median (IQR, N) | 136.5 (72-228.75, 18) | 83.5 (80.25-90.75, 10) |
| **Cytoreductive treatment** |  |  |
| - TKI, % (N) | 56 (10/18) | 25 (3/12) |
| - AlloHCT, % (N) | 100 (18/18) | 0 (0/12) |
| - Poly-chemotherapy, % (N) | 61 (11/18) | 67 (8/12) |
| - 2-CDA, % (N) | 11 (2/18) | 0 (0/12) |
| - IFN-alpha, % (N) | 0 (0/18) | 0 (0/12) |
| - HU, % (N) | 6 (1/18) | 17 (2/12) |
| **Survival after SM diagnosis** |  |  |
| - Deaths, n/N | 6/18 | 8/12 |
| - 1-year OS, % (95% CI)* | 100 (100-100) | 64 (41-100) |
| - 2-year OS, % (95% CI)* | 94 (84-100) | 26 (8-82) |
| - 5-year OS, % (95% CI)* | 50 (27-93) | 13 (2-78) |
| **Allo-HCT specific variables (events censored 60 months after SM diagnosis)**** | | |
| **Time from AdvSM to allo-HCT,** median (IQR, N) | 4.1 (3.39-6.08, 18) |  |
| **Karnofsky performance status at time of allo-HCT,** median (IQR, N) | 90 (85-100, 17) |  |
| **IPSM Score at time of allo-HCT,** median (IQR, N) | 3 (2-3, 18) |  |
| - group 1, % (N) | 17 (3/18) |  |
| - group 2, % (N) | 17 (3/18) |  |
| - group 3, % (N) | 50 (9/18) |  |
| - group 4, % (N) | 17 (3/18) |  |
| **Conditioning** |  |  |
| - Myeloablative, % (N) | 65 (11/17) |  |
| - Reduced intensity, % (N) | 35 (6/17) |  |
| - CR/PR before Allo-HCT, % (N) | 56 (10/18) |  |
| **Outcome after allo-HCT** |  |  |
| - Relapse, % (N) | 44 (8/18) |  |
| - Death, % (N) | 33 (6/18) |  |
| - Relapse related, % (N) | 33 (2/6) |  |
| - allo-HCT related, % (N) | 50 (3/6) |  |
| - Other cause of death, % (N) | 17 (1/6) |  |
| **Survival after allo-HCT** |  |  |
| - 1-year OS, % (95% CI) | 83 (68-100) |  |
| - 2-year OS, % (95% CI) | 83 (68-100) |  |
| - 5-year OS, % (95% CI) | 53 (30-95) |  |
| **Relapse after allo-HCT** |  |  |
| - 1-year PFS, % (95% CI) | 83 (68-100) |  |
| - 2-year PFS, % (95% CI) | 78 (61-100) |  |
| - 5-year PFS, % (95% CI) | 45 (24-84) |  |
| Abbreviations: 2-CdA, Cladribine; AdvSM, advanced systemic mastocytosis; AHN, associated hematologic neoplasm; allo-HCT, allogeneic hematopoietic cell transplantation; AP, alkaline phosphatase; ASM, aggressive systemic mastocytosis; IFN-α, interferon alpha; IQR, interquartile range; IPSM, international prognostic scoring system in mastocytosis; LDH, lactate dehydrogenase; m, male; MCL, mast cell leukemia; n, number; NA, not available; S/A/R, *SRSF2*/*ASXL1*/*RUNX1*; SM-AHN, systemic mastocytosis with an associated hematologic neoplasm; SM-AML, systemic mastocytosis with an associated acute myeloid leukemia; TKI, tyrosine kinase inhibitor (includes midostaurin, avapritinib, imatinib, and investigational TKIs); WBC, white blood cells; WHO, world health organization.  * OS estimates from SM diagnosis are descriptive only. Patients must survive until transplantation to be classified as transplanted, so this comparison is subject to immortal-time bias.  ** Administrative censoring was performed to make results comparable to the results derived from our complete case cohort with 419 patients. The data used for this table is in agreement with the data for the 30 SM-AML patients included in our allo-HCT and TKI treatment efficacy analysis. | | |

# Supplementary Figures

## Supplementary Figure 1: Kaplan-Meier survival estimates stratified by prognostic factors for overall survival in the complete-case AdvSM cohort.

Kaplan-Meier estimates of overall survival from AdvSM diagnosis in the complete-case cohort (*n = 419*), stratified by: **(A)** Full complete case cohort, **(B)** AdvSM subtype, **(C)** IPSM risk group, **(D)** *KIT* D816V mutation status, **(E)** WHO performance status, and **(F)** sex.

Abbreviations: AdvSM, advanced systemic mastocytosis; CI, confidence interval; IPSM, **International Prognostic Scoring System in Mastocytosis;** OS, overall survival; TKI, tyrosine kinase inhibitor; WHO, world health organization.

## Supplementary Figure 2: Multivariate sensitivity analysis including TKI treatment instead of TKI response.

A sensitivity analysis substituting response to TKI treatment with any TKI treatment (start of first TKI treatment) as a time-dependent covariate in the complete-case cohort (n = *419*, Allo-HCT *n = 63*, death *n = 174*).

The forest plots show hazard ratios (HRs) for death (HR>1 indicates increased risk). Allo-HCT treatment and TKI were modeled as time-dependent covariates. Non-time-dependent prognostic variables include male sex, AdvSM subtype (other AHN vs. comparator), KIT D816V (positive vs. negative), WHO performance status (per 1-point increase), and IPSM risk group (per 1-category increase). Center experience with AdvSM patients (HR per 10 patients treated) and year of diagnosis (HR per decade since first AdvSM diagnosis among study participants) are also included.

Abbreviations: AdvSM, advanced systemic mastocytosis; Allo-HCT, allogeneic hematopoietic stem cell transplantation; ASM, aggressive systemic mastocytosis; CI, confidence interval; HR, hazard ratio; IPSM, **International Prognostic Scoring System in Mastocytosis;** NA, not available; MCL, mast cell leukemia; n, number; SM-AHN, systemic mastocytosis with an associated hematologic neoplasm; SM-AML, systemic mastocytosis with an associated acute myeloid leukemia; TKI, tyrosine kinase inhibitor; WHO, world health organization.

## Supplementary Figure 3: Study design and patient selection.

Flowchart showing inclusion and exclusion of mastocytosis patients from the ECNM registry (*n = 5465*). Patients with CM/MIS (*n* *= 2068*), ISM/BMM/SSM (n = 2697), and MCS (*n = 4*) were excluded. After removal of patients with insufficient FU after diagnosis (*n = 65*), the remaining patients with AdvSM (*n = 631*; including *n = 69* who received allo-HCT) were included for descriptive analyses and assessment of prognostic factors for progression-free and overall survival after allo-HCT. Patients with WHO performance status >3, or missing covariates, age <18 or >75 years were further excluded, resulting in a complete-case cohort (*n* *= 419*; including *n* *= 63* who received allo-HCT) that was used for uni- and multivariate analyses to assess the impact of allo-HCT and TKI treatment on survival in AdvSM.

Abbreviations: AdvSM, advanced systemic mastocytosis; Allo-HCT, allogeneic hematopoietic cell transplantation; BMM, bone marrow mastocytosis; CM, cutaneous mastocytosis; ECNM, european competence network on mastocytosis; FU, follow-up; ISM, indolent systemic mastocytosis; MCS, mast cell sarcoma; MIS, mastocytosis in the skin; PS, performance status; SSM, smoldering systemic mastocytosis; TKI, tyrosine kinase inhibitor; WHO, world health organization.

## Supplementary Figure 4: Kaplan-Meier survival estimates stratified by prognostic factors for overall survival in the full AdvSM cohort.

Kaplan-Meier estimates of overall survival from AdvSM diagnosis in the full AdvSM cohort (*n = 631*). **(A)** Full cohort, and stratified by: **(B)** *KIT* D816V mutation status, **(C)** WHO performance status, **(D)** sex. This cohort included all patients with AdvSM regardless of age, WHO performance status, or covariate completeness, in contrast to the complete-case cohort (*n = 419)* used for the multivariate analysis in **figure 1**.

Abbreviations: AdvSM, advanced systemic mastocytosis; CI, confidence interval; IPSM, **International Prognostic Scoring System in Mastocytosis; NA, not available;** OS, overall survival; TKI, tyrosine kinase inhibitor; WHO, world health organization.

## Supplementary Figure 5: Prognostic factors for overall survival in AdvSM.

Kaplan–Meier estimates of overall survival (OS) for the primary cohort (*n = 631*) stratified by **(A)** AdvSM subtype and stratified by **(B)** IPSM risk group 1-4. **(C)** Forest plot from the multivariate analysis of the complete-case cohort (*n = 419*) showing hazard ratios (HRs) for death (HR>1 indicates increased risk). Allo-HCT treatment and tyrosine kinase inhibitor (TKI) response were modeled as time-dependent covariates. Non-time-dependent prognostic variables include male sex, AdvSM subtype (other AHN vs. comparator), KIT D816V (positive vs. negative), WHO performance status (per 1-point increase), and IPSM risk group (per 1-category increase). Center experience with AdvSM patients (HR per 10 patients treated) and year of diagnosis (HR per decade since first AdvSM diagnosis among study participants) are also included.

Abbreviations: AdvSM, advanced systemic mastocytosis; AHN, associated hematologic neoplasm; allo-HCT, allogeneic hematopoietic cell transplantation; ASM, aggressive systemic mastocytosis; CI, confidence interval; HR, hazard ratio; IPSM, international prognostic scoring system in mastocytosis; MCL, mast cell leukemia; n, number; NA, not available; No, number of patients; OS, overall survival; SM-AHN, systemic mastocytosis with an associated hematological neoplasm; SM-AML, systemic mastocytosis with an associated acute myeloid leukemia; TKI, tyrosine kinase inhibitor; WHO, world health organization.

## Supplementary Figure 6: Overall and progression-free survival and competing risk analysis in AdvSM patients after allo-HCT.

Kaplan-Meier estimates of PFS and OS and cumulative incidence of competing mortality risks in AdvSM patients who received allo-HCT. **(A)** PFS for the allo-HCT cohort (n = 67). **(B)** OS for the allo-HCT cohort (n = 69). **(C)** Cumulative incidence of RRM, TRM, and other causes of death. Survival is calculated from the time of allo-HCT.

Abbreviations: AdvSM, advanced systemic mastocytosis; allo-HCT, allogeneic hematopoietic stem cell transplantation; CI, confidence interval; OS, overall survival; PFS, progression-free survival; RRM, relapse-related mortality; TRM, transplant-related mortality.

## Supplementary Figure 7: Overall and progression-free survival and competing risk analysis in AdvSM patients after allo-HCT stratified by AdvSM subtype.

Kaplan-Meier estimates of progression-free survival (PFS) and overall survival (OS) and cumulative incidence of competing mortality risks in AdvSM subtypes after allo-HCT. **(A)** PFS for the allo-HCT cohort stratified by IPSM risk group (n = *63*). **(B)** OS for the allo-HCT cohort stratified by IPSM risk group (n = *65*). **(C)** PFS for the allo-HCT cohort stratified by AdvSM subtype (n = *69*). **(D)** OS for the allo-HCT cohort stratified by AdvSM subtype (n = *69*). **(E)** Cumulative incidence of RRM, TRM, and other causes of death stratified by AdvSM subtype. Survival is calculated from the time of allo-HCT.

Abbreviations: AdvSM, advanced systemic mastocytosis; **AHN, associated hematologic neoplasm;** allo-HCT, allogeneic hematopoietic stem cell transplantation; CI, confidence interval; **ASM, aggressive systemic mastocytosis; MCL, mast cell leukemia;** OS, overall survival; PFS, progression-free survival; RRM, relapse-related mortality; **SM-AML, systemic mastocytosis with associated acute myeloid leukemia;** TRM, transplant-related mortality.

# Supplementary References

1.	Valent, P. *et al.* The Data Registry of the European Competence Network on Mastocytosis (ECNM): Set Up, Projects, and Perspectives. *J Allergy Clin Immunol Pract* **7**, 81–87 (2019).

2.	Valent, P. *et al.* Updated Diagnostic Criteria and Classification of Mast Cell Disorders: A Consensus Proposal. *Hemasphere* **5**, e646 (2021).

3.	Valent, P. *et al.* Advances in the Classification and Treatment of Mastocytosis: Current Status and Outlook toward the Future. *Cancer Res* **77**, 1261–1270 (2017).

4.	Valent, P. *et al.* Diagnostic criteria and classification of mastocytosis: a consensus proposal. *Leuk Res* **25**, 603–625 (2001).

5.	Valent, P. *et al.* Standards and standardization in mastocytosis: consensus statements on diagnostics, treatment recommendations and response criteria. *Eur J Clin Invest* **37**, 435–453 (2007).

6.	Arber, D. A. *et al.* International Consensus Classification of Myeloid Neoplasms and Acute Leukemias: integrating morphologic, clinical, and genomic data. *Blood* **140**, 1200–1228 (2022).

7.	Valent, P., Akin, C. & Metcalfe, D. D. Mastocytosis: 2016 updated WHO classification and novel emerging treatment concepts. *Blood* **129**, 1420–1427 (2017).

8.	Sperr, W. R. *et al.* International prognostic scoring system for mastocytosis (IPSM): a retrospective cohort study. *Lancet Haematol* **6**, e638–e649 (2019).

9.	Jawhar, M. *et al.* MARS: Mutation-Adjusted Risk Score for Advanced Systemic Mastocytosis. *J Clin Oncol* **37**, 2846–2856 (2019).
